# Supplementary material for: Genomic regions under selection in the feralization of the dingoes
Source: Nat Commun. 2020 Feb 3;11:671. doi: 10.1038/s41467-020-14515-6 (PMC6997406; doi:10.1038/s41467-020-14515-6)
Supplement: Supplementary file 1 — Supplementary Information [file 41467_2020_14515_MOESM1_ESM.pdf]

Supplementary Information

Genomic regions under selection in the feralization of the dingoes

Zhang, Wang, Ma et al.

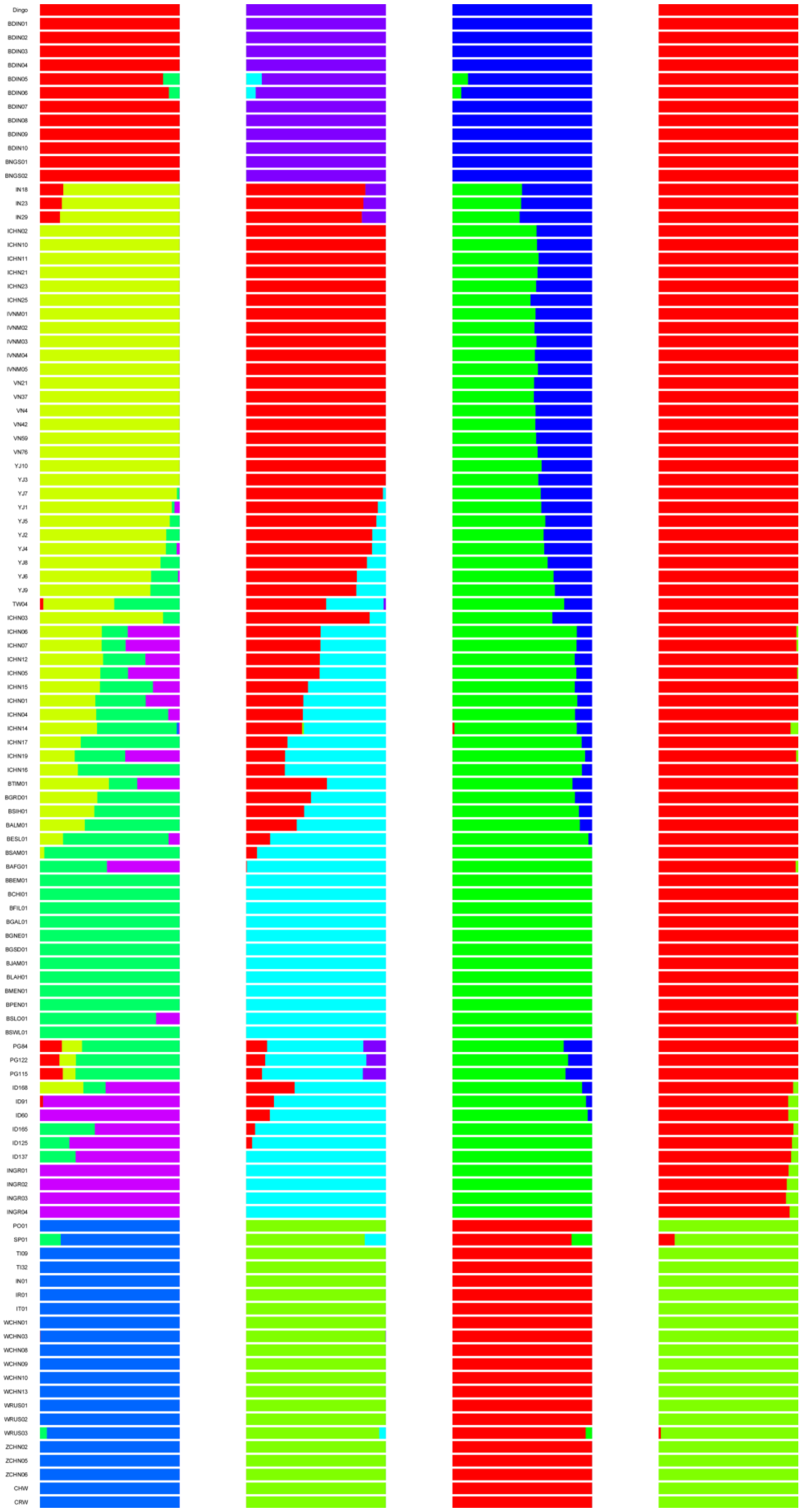

Supplementary Figure 1. Population structure for k = 2 to k = 5 with detailed sample labels.

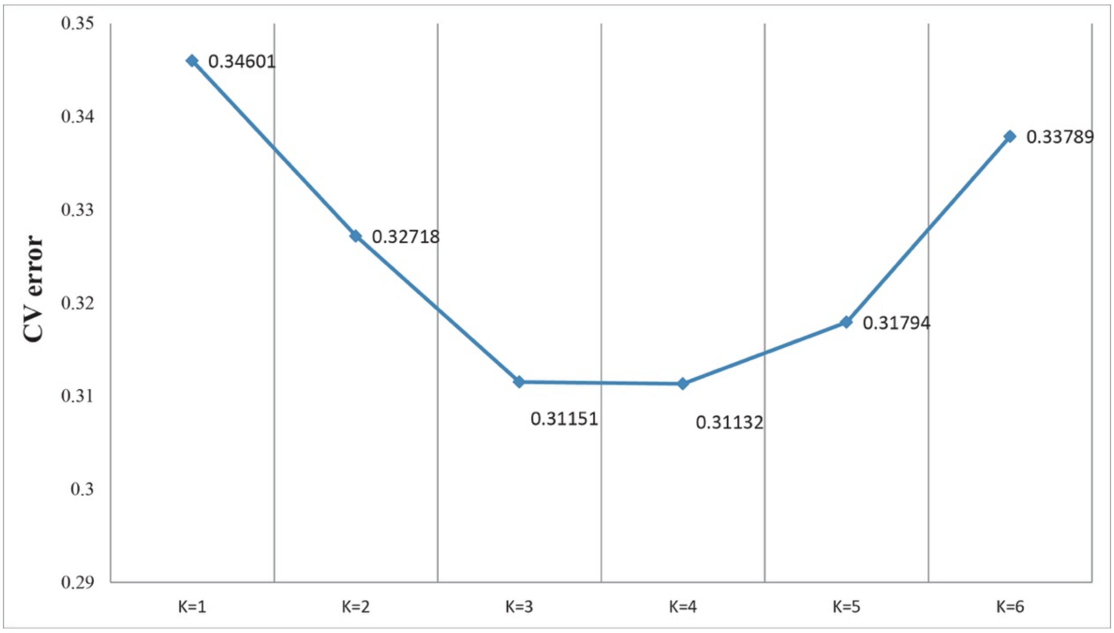

Supplementary Figure 2. Cross-validation plot for the ADMIXTURE analyses (Supplementary Figure 1). K ranges from 1 to 6.

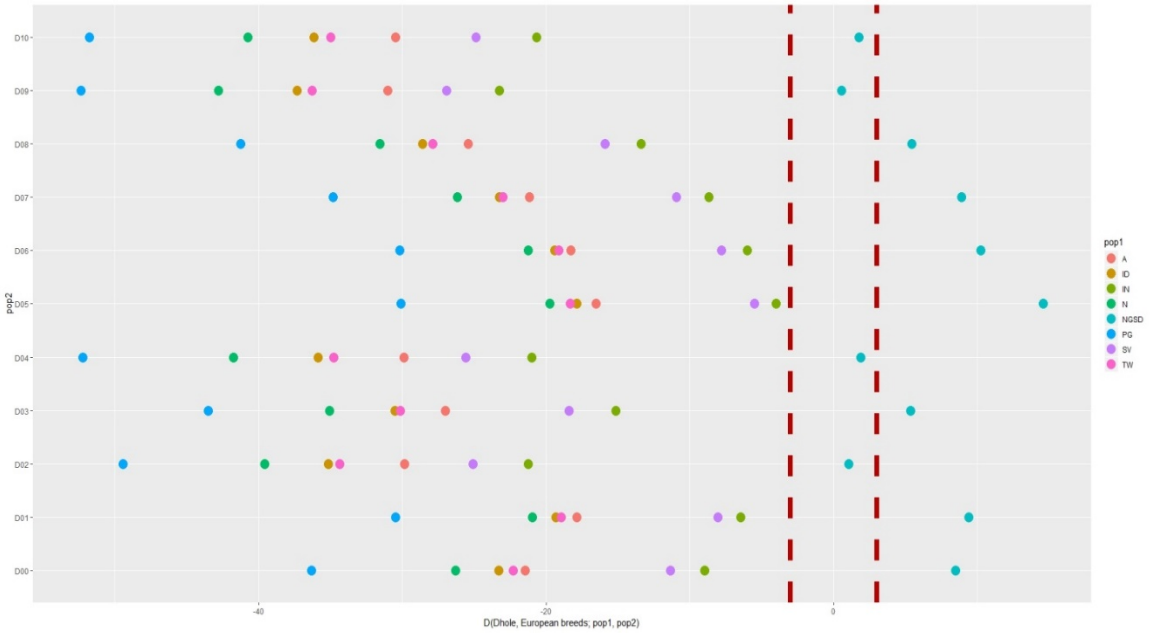

Supplementary Figure 3. D (Dhole,European breed;Pop1,Pop2), with the Z-score given on the X axis. NGSD, New Guinea Singing Dogs; IN, Indonesian village dogs; SV, indigenous dogs from southern China; TW, Taiwan indigenous dog; N, indigenous dogs from north China; ID, Indian village dogs; A, African village dogs. The dingoes are Pop2. Z<-3 for all Pop1 groups except the NGSDs.

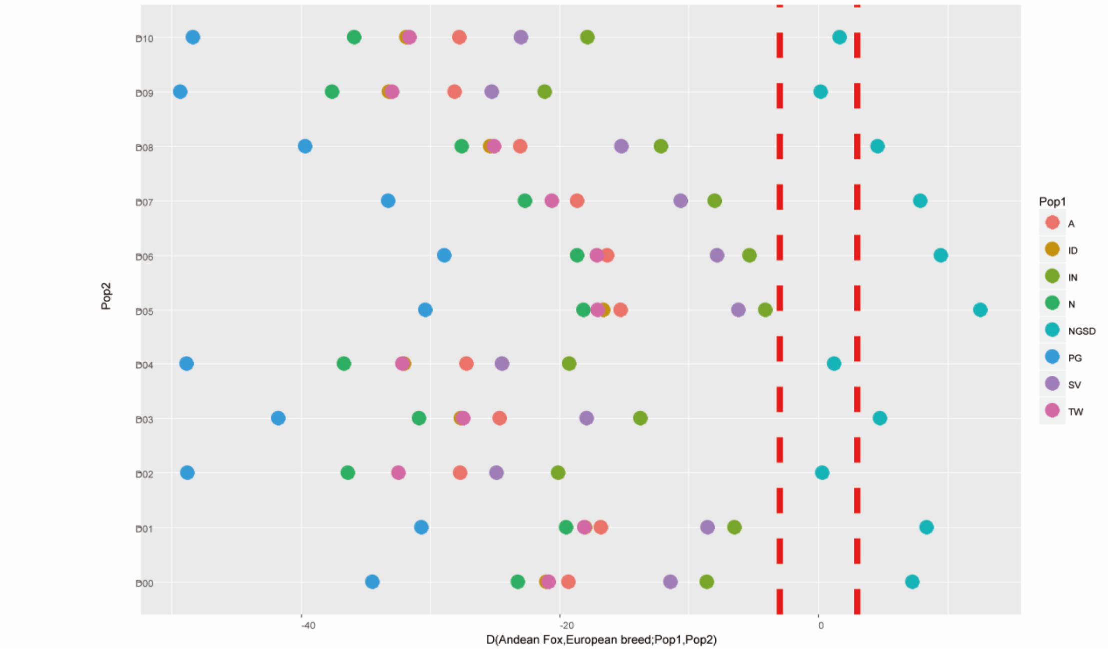

Supplementary Figure 4. D (Fox,European breed;Pop1,Pop2), with the Z-score given on the X axis. NGSD, New Guinea Singing Dogs; IN, Indonesian village dogs; SV, indigenous dogs from southern China; TW, Taiwan indigenous dog; N, indigenous dogs from north China; ID, Indian village dogs; A, African village dogs. The dingoes are Pop2. Z<-3 for all Pop1 groups except the NGSDs.

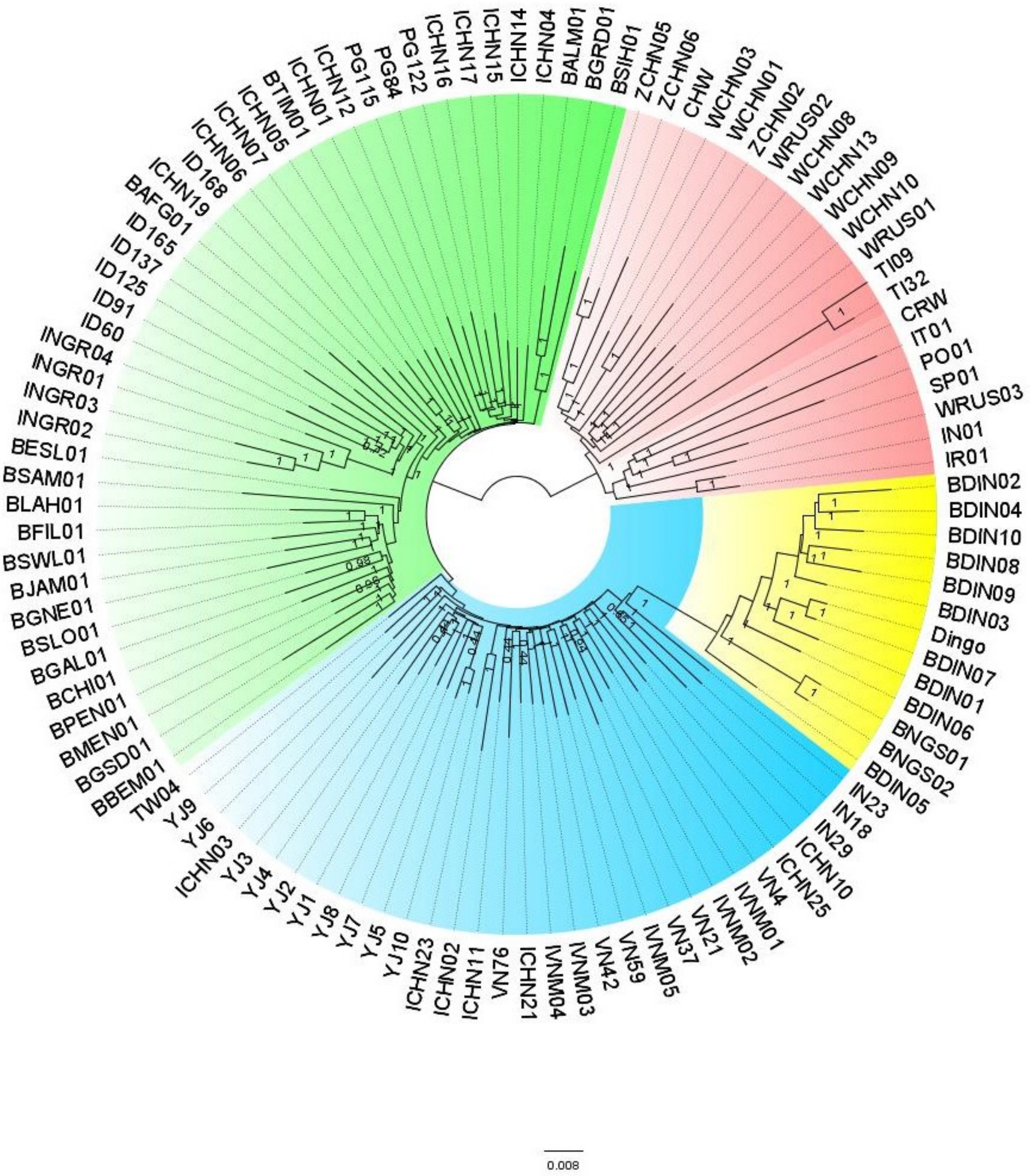

Supplementary Figure 5. Neighbor-Joining phylogenetic tree for all 109 individuals, identical with the tree in Figure 1E, with detailed sample labels, and node labels indicating bootstrap values. The 109 individuals split into three distinct clades: (1) wolves (orange), (2) indigenous dogs from Indonesia, southern East Asia and Taiwan village dog (blue), including the clade of dingoes and NGSDs (yellow), (3) village dogs and dog breeds from all other regions (green).

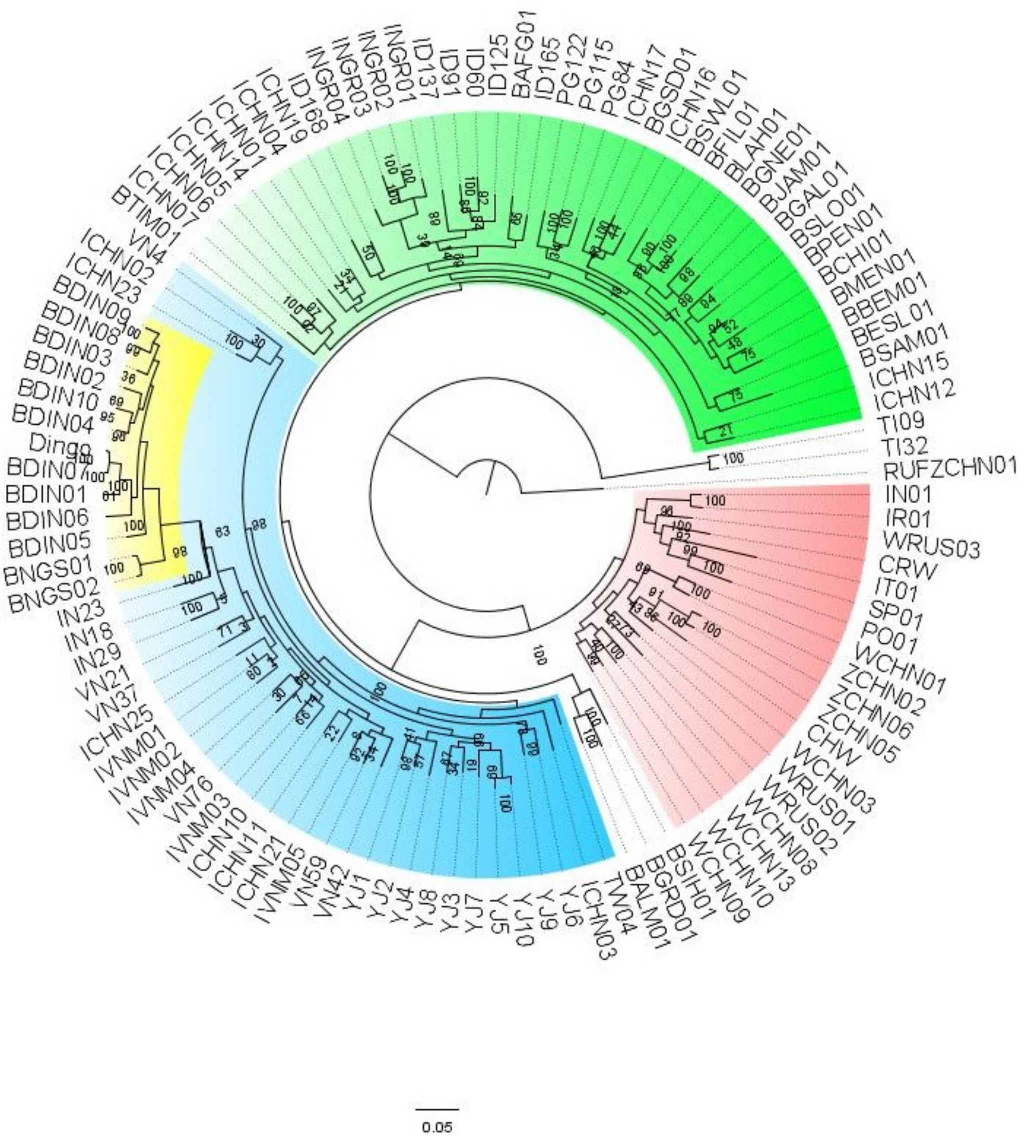

Supplementary Figure 6. Maximum-likelihood phylogenetic tree for all the 109 individuals and also an outgroup (Dhole (RUFZCHN01)), with detailed sample labels, and node labels indicating bootstrap values. The topology is consistent with the NJ tree, except five individuals that dissociate from the main clades: Tibetan wolves (TI09, TI32) and three arctic breeds: Siberian Huskey (BSIH01), Greenland dog (BGRD01) and Alaskan Malamute (BALM01).

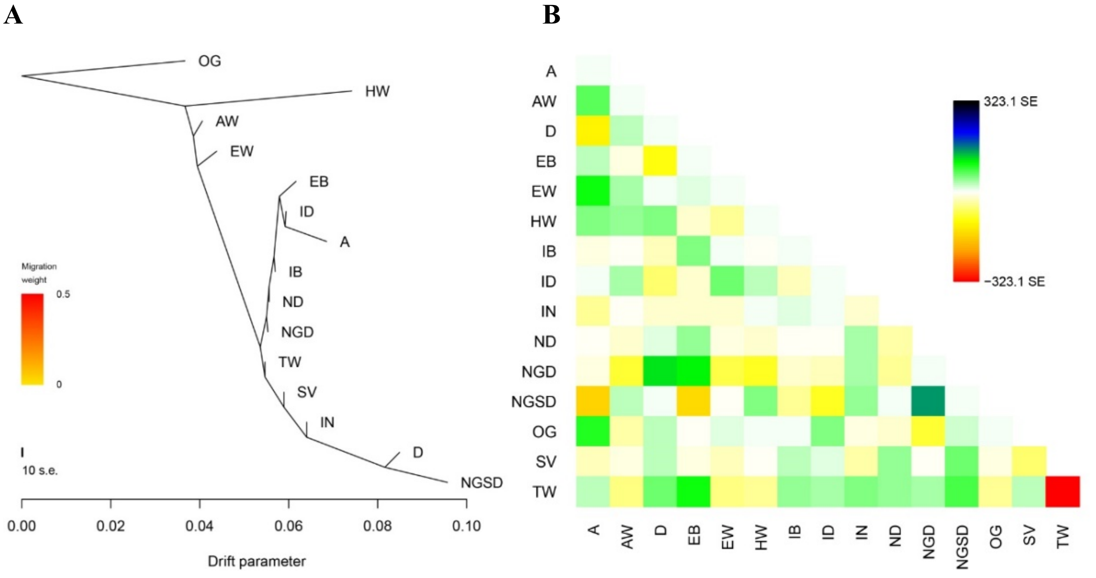

Supplementary Figure 7. TreeMix for all the 109 individuals without migration (A) and matrix of residuals (B). The matrix of residuals stand for how the estimated genetic relationship between each pair of groups fit the model. A high residual indicates that the pair does not fit the model and may have had an admixture event. We find one candidate admixture event: between the dingo/NGSD and the Papua New Guinea village dogs. D, dingoes; NGSD, New Guinea Singing Dogs; IN, Indonesian village dogs ; SV, indigenous dogs from southern China; TW, Taiwan indigenous dog; ND, indigenous dogs from north China; IB, intermediate breeds; EB, European breeds; NGD, Papua New Guinea village dogs; ID, Indian village dogs; A, African village dogs; AW, Asian wolves; EW, European wolf; HW, Tibetan wolves; OG, Outgroup (Dhole).

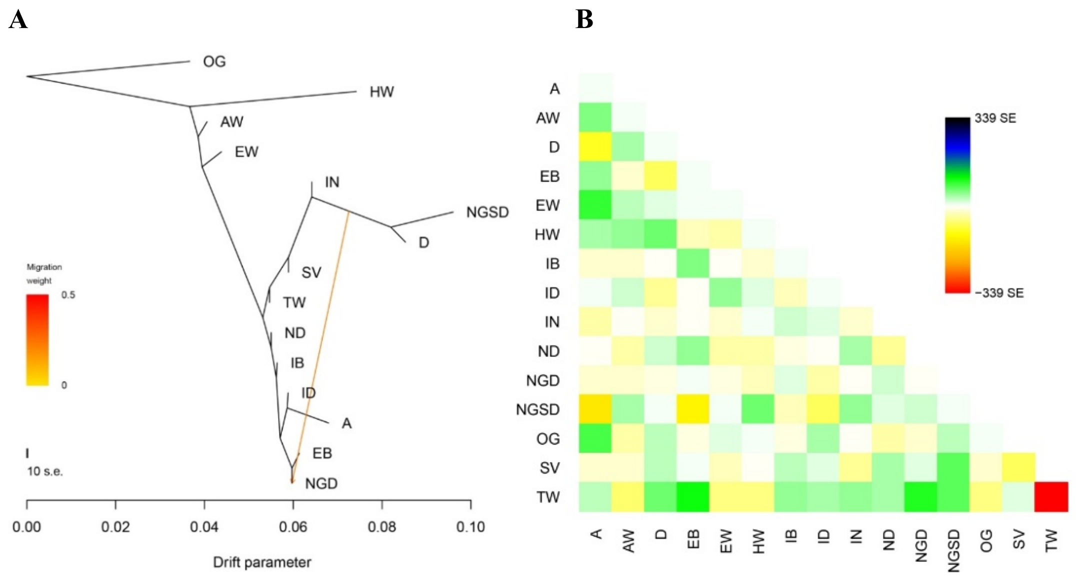

Supplementary Figure 8. TreeMix for all the 109 individuals with one migration (A) and matrix of residuals (B). The single migration event is from the dingo/NGSD lineage to the Papua New Guinea village dog clade, resulting in that all the residuals are low. D, dingoes; NGSD, New Guinea Singing Dogs; IN, Indonesian village dogs ; SV, indigenous dogs from southern China; TW, Taiwan indigenous dog; ND, indigenous dogs from north China; IB, intermediate breeds; EB, European breeds; NGD, Papua New Guinea village dogs; ID, Indian village dogs; A, African village dogs; AW, Asian wolves; EW, European wolf; HW, Tibetan wolves; OG, Outgroup (Dhole).

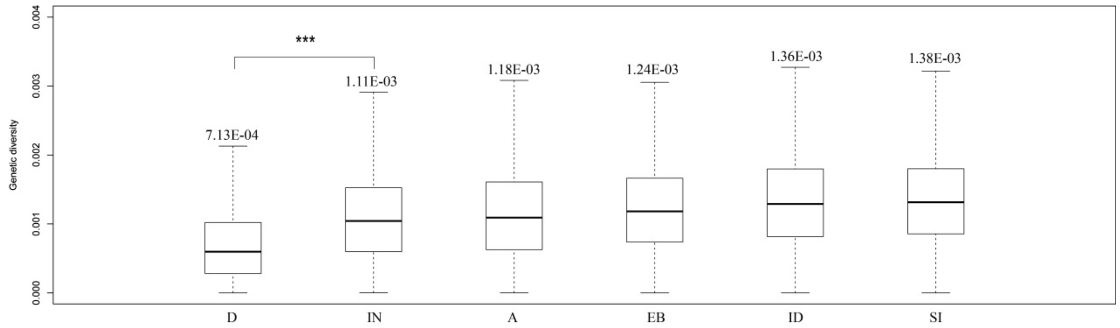

Supplementary Figure 9. Genetic diversity for the six dog groups. D, 11 dingoes from Australia; IN, 3 Indonesian indigenous dogs; A, 4 Nigerian dogs; EB, 11 European breeds; ID, 6 Indian village dogs; SI, 27 indigenous dogs from southern China and Vietnam; (***, $p<2.2e-16$). We estimated nuclear diversity using the parameter θ_π. The parameter $\theta\pi$ was calculated from a 40 kb windows with step sizes of 20 kb across the genome using VCFtools (--window-pi). Mean values: D, 7.13E-04; IN, 1.11E-03; A, 1.18E-03; EB, 1.24E-03; ID, 1.36E-03; SI, 1.38E-03. Source data are provided as a Source Data file.

A

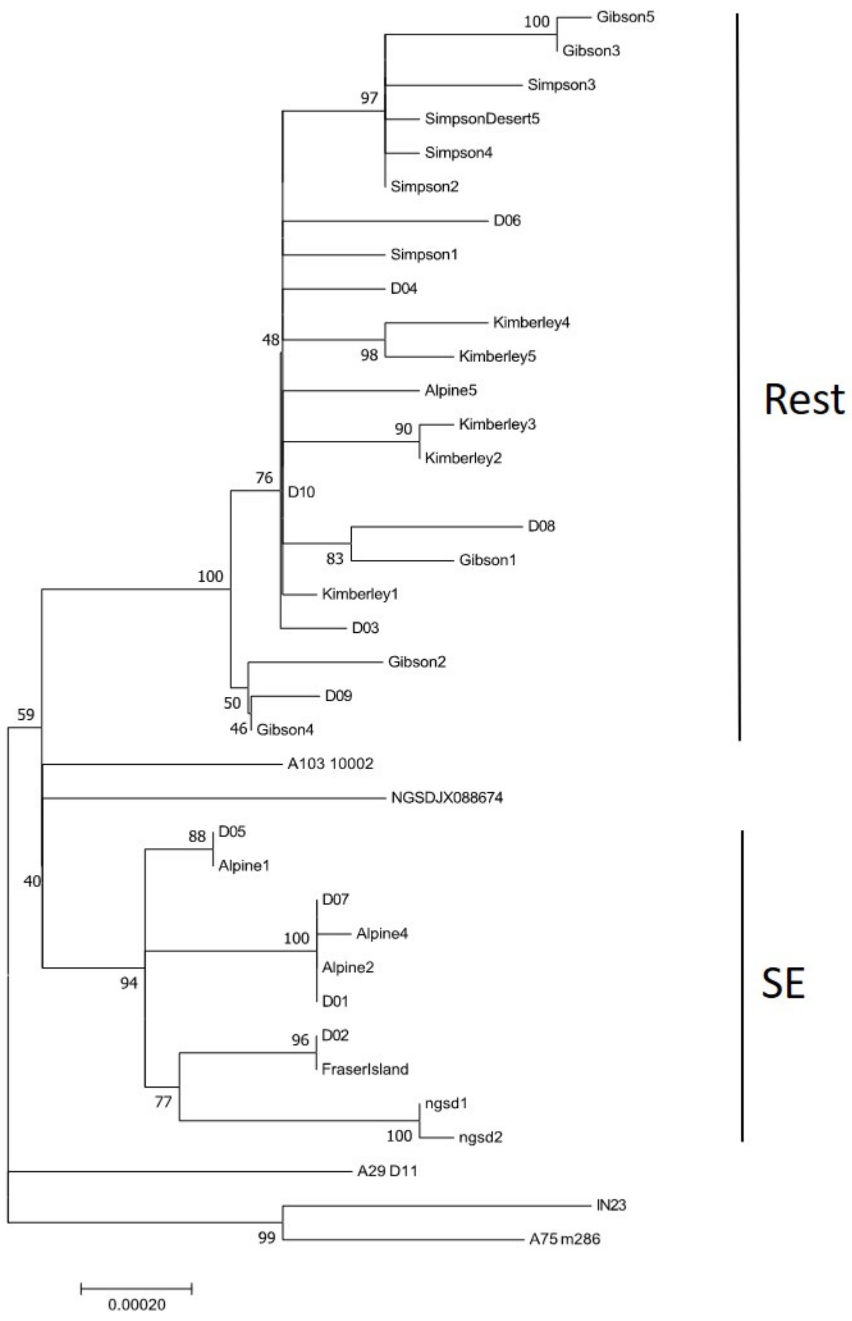

Supplementary Figure 10. mtDNA neighbor joining tree of all dingoes/NGSDs and four most closely related dogs.

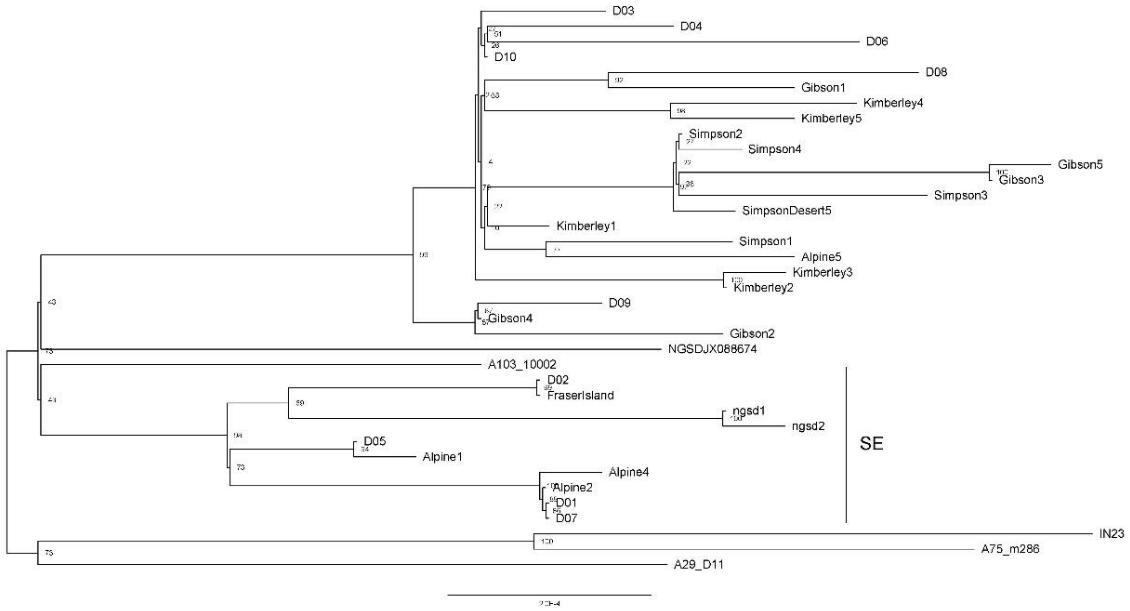

Supplementary Figure 11. mtDNA maximum likelihood tree of all dingoes/NGSDs and four most closely related dogs. To study the detailed phylogeny among dingoes and NGSDs we created a sub-dataset including all individuals in the dingo/NGSD branch and the three most closely related dogs (yellow box in Fig.3A), and constructed new phylogenetic trees.

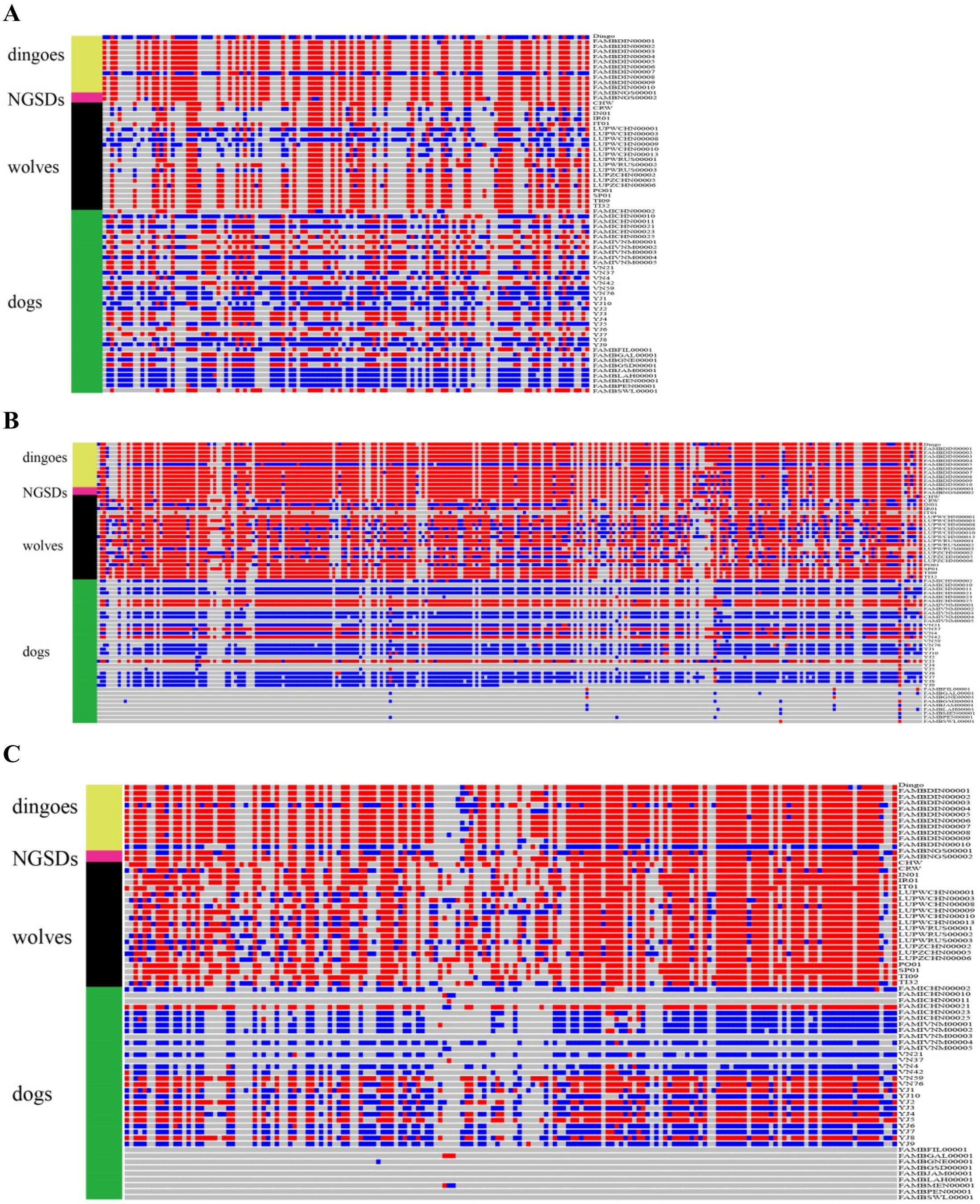

Supplementary Figure 12. Genotype matrix for three loci (including the four genes *ARHGEF7, Prss37, TAS2R5 and SLC5A1)* for 70 canine samples. Groupings are indicated on the left of the plot: dingoes (yellow), NGSDs (pink), wolves (black), indigenous dogs from southern China and European breeds (green) and. In the plots, colors indicate the genotype: sites homozygous for the reference (0/0; colored gray), homozygous for the alternative variant (1/1; red) and heterozygous sites (0/1; blue). Each column represents a single SNP, and each row a sample.

A) Chr22: 59220001-59260001 (*ARHGEF7*), 128 SNPs.

B) Chr16: 7320000- 7360000 (*Prss37* and *TAS2R5*), 277 SNPs.

C) Chr26: 24940000-24960000 (*SLC5A1*), 175 SNPs.

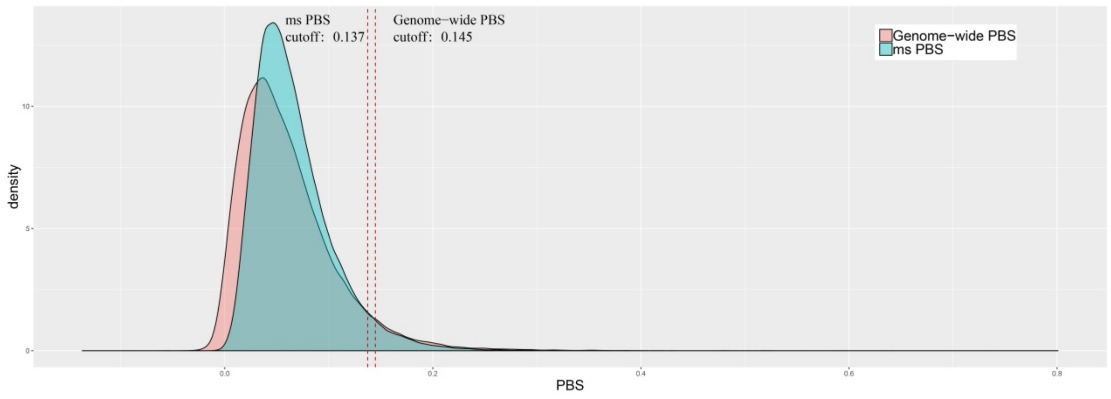

Supplementary Figure 13. Distribution of Genome-wide (red) and simulated (green) PBS1 values in non-overlapping 20 kb genomic windows. The simulated data top 5% (0.13736) is indicated as a red dotted line and the minimum value of Genome-wide top 5% PBS1 is 0.14476.

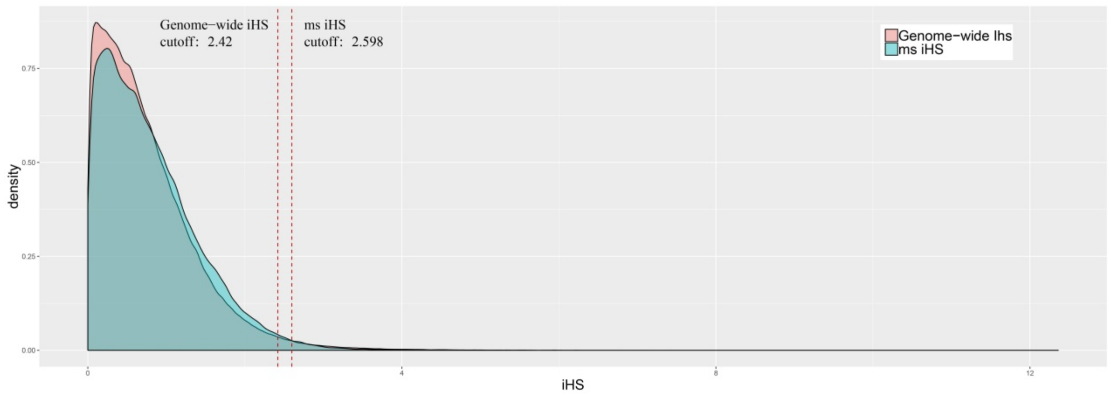

Supplementary Figure 14. Distribution of Genome-wide (red) and simulated (green) iHS absolute values. The first percentile rank of simulated data (2.5983) was used as a threshold to identify high iHS values.

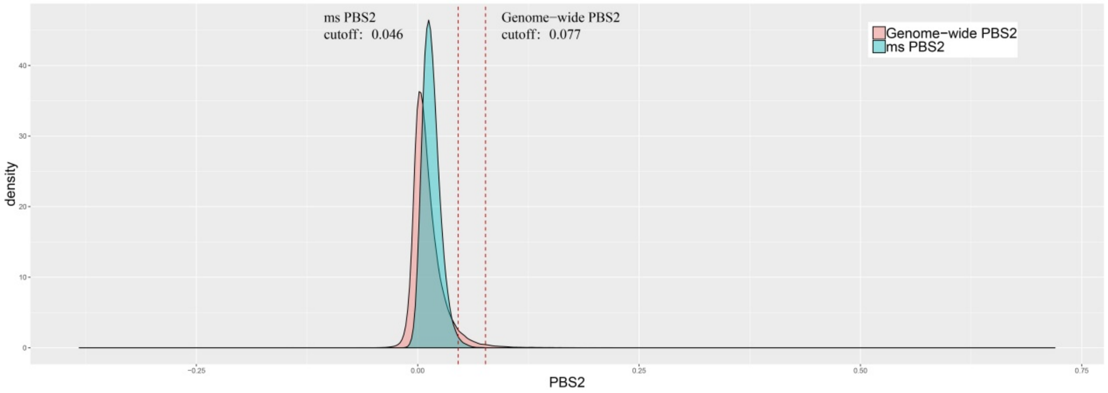

Supplementary Figure 15. Distribution of Genome-wide (red) and simulated (green) PBS2 values in non-overlapping 20 kb genomic windows. The simulated data top 1% (0.0457) is indicated as a red dotted line and the minimum value of Genome-wide top 1% PBS is 0.07660.

Supplementary Table 1. Outgroup f3-statistics analysis.

(dingo,pop2;outgroup)		
pop2	f_3	Z_score
NGSDs	22.060072	45.08
Indonesian village dogs	20.62551	44.795
Indigenous dogs from southern China	20.161008	45.056
Breeds	20.082663	45.174
Indigenous dogs from north China	19.933636	45.15
Indian village dogs	19.622791	44.97
African village dogs	19.469508	44.913
Wolves	16.432468	46.682

Supplementary Table 2. Samples used for the first G-phocs analysis.

population	random1	random2	random3
SV dog	ICHN03, YJ7, ICHN02	YJ6, YJ10, IVNM03	YJ7, YJ3, IVNM05
IN dog	IN18, IN23, IN29	IN18, IN23, IN29	IN18, IN23, IN29
dingo	D01, D02, D09	D02, D07, D08	D03, D04, D08

SV dog is Indigenous dog from southern China, and IN dog is Indonesian village dog.

Supplementary Table 3. Samples used for the second round of G-phocs analysis.

population	random1	random2	random3
SV dog	ICHN03, YJ7, ICHN02	YJ6, YJ10, IVNM03	YJ7, YJ3, IVNM05
IN dog	IN18, IN23, IN29	IN18, IN23, IN29	IN18, IN23, IN29
dingo	D01, D02, D06	D02, D07, D08	D03, D04, D05

SV dog is Indigenous dog from southern China, and IN dog is Indonesian village dog.

Supplementary Table 4. The 17 candidate windows under selection in dingoes and more similar to grey wolves than to domestic dogs. Source data are provided as a Source Data file.

Chr	Start	End	PBS1 value	PBS2 value	Proportion of sites above iHS threshold	Gene
chr10	7740001	7760001	0.189038	0.14511	0.388888889	
chr10	13800001	13820001	0.257726	0.088805	0.6	*TRHDE*
chr10	17020001	17040001	0.312135	0.124486	0.317460317	*TUBGCP6, SELENOO ENSCAFG00000000697, TRABD*
chr10	17040001	17060001	0.312135	0.094363	0.350877193	*PANX2*
chr13	25000001	25020001	0.187243	0.087072	0.452380952	
chr14	21500001	21520001	0.170606	0.106193	0.350877193	
chr15	60140001	60160001	0.202368	0.084002	0.525252525	
chr16	7300001	7320001	0.307687	0.137438	0.653846154	
chr16	7340001	7360001	0.328073	0.127024	0.461538462	*PRSS37, ENSCAFG00000003879*
chr16	7400001	7420001	0.276338	0.102698	0.5	*RF00026, TAS2R5*
chr18	4940001	4960001	0.210151	0.099769	0.725806452	
chr18	4960001	4980001	0.195234	0.101103	0.848484848	
chr22	59220001	59240001	0.149967	0.084041	0.358024691	*ARHGEF7*
chr23	26980001	27000001	0.196895	0.083557	0.409090909	*ANKRD28*
chr26	24940001	24960001	0.231977	0.113768	0.325	*SLC5A1*
chr34	5560001	5580001	0.156079	0.087471	0.322580645	
chr9	17400001	17420001	0.241287	0.190799	1	
