# Supplementary material for: Genomic regions under selection in the feralization of the dingoes
Source: Nat Commun. 2020 Feb 3;11:671. doi: 10.1038/s41467-020-14515-6 (PMC6997406; doi:10.1038/s41467-020-14515-6)
Supplement: Supplementary file 3 — Reporting Summary [file 41467_2020_14515_MOESM3_ESM.pdf]

Corresponding author(s): Prof. Ya-Ping Zhang (zhangyp@mail.kiz.ac.cn) (Kunming Institute of Zoology)

Last updated by author(s): Dec 11, 2019

Reporting Summary

Nature Research wishes to improve the reproducibility of the work that we publish. This form provides structure for consistency and transparency in reporting. For further information on Nature Research policies, see Authors & Referees and the Editorial Policy Checklist.

Statistics

For all statistical analyses, confirm that the following items are present in the figure legend, table legend, main text, or Methods section.

n/a	Confirmed	
☐	☒	The exact sample size (*n*) for each experimental group/condition, given as a discrete number and unit of measurement
☐	☒	A statement on whether measurements were taken from distinct samples or whether the same sample was measured repeatedly
☐	☒	The statistical test(s) used AND whether they are one- or two-sided<br>*Only common tests should be described solely by name; describe more complex techniques in the Methods section.*
☐	☒	A description of all covariates tested
☐	☒	A description of any assumptions or corrections, such as tests of normality and adjustment for multiple comparisons
☐	☒	A full description of the statistical parameters including central tendency (e.g. means) or other basic estimates (e.g. regression coefficient) AND variation (e.g. standard deviation) or associated estimates of uncertainty (e.g. confidence intervals)
☐	☒	For null hypothesis testing, the test statistic (e.g. *F*, *t*, *r*) with confidence intervals, effect sizes, degrees of freedom and *P* value noted<br>*Give P values as exact values whenever suitable.*
☐	☒	For Bayesian analysis, information on the choice of priors and Markov chain Monte Carlo settings
☐	☒	For hierarchical and complex designs, identification of the appropriate level for tests and full reporting of outcomes
☒	☐	Estimates of effect sizes (e.g. Cohen's *d*, Pearson's *r*), indicating how they were calculated

Our web collection on statistics for biologists contains articles on many of the points above.

Software and code

Policy information about availability of computer code

Data collection	The raw sequence data from this study have been submitted to the GSA (http://gsa.big.ac.cn/) under accession CRA000200 for raw data of genomes.
Data analysis	We used these tools in data analysis : Burrows-Wheeler Aligner (BWA) 0.7.10, picard tools 1.87, GenomeAnalysisTK(GATK) 2.5-2, VCFtools 0.1.9.0, plink 1.07, smartPCA(EIG6.0.1), MEGA 7, G-PhoCS 1.3, TRACER 1.5, selscan 1.1.0, qpDstat, qp3pop, ms,topGO R package.

For manuscripts utilizing custom algorithms or software that are central to the research but not yet described in published literature, software must be made available to editors/reviewers. We strongly encourage code deposition in a community repository (e.g. GitHub). See the Nature Research guidelines for submitting code & software for further information.

Data

Policy information about availability of data

All manuscripts must include a data availability statement. This statement should provide the following information, where applicable:

- Accession codes, unique identifiers, or web links for publicly available datasets
- A list of figures that have associated raw data
- A description of any restrictions on data availability

This project has also been deposited at the National Center for Biotechnology Information (NCBI) Sequence Read Archive database. The accession number is PRJNA593363 (SRP234866). We have used downloaded data from published articles, and their accession number is SRA307300, SRP044399, SRP035294, SRP062184, SRP062060 and SRP058219. The dog reference genome is Canfam3.The source data underlying Figs 1b, c, 4a–c, 5 and Supplementary Fig 9 and Supplementary Table 4 are provided as a Source Data file.

Field-specific reporting

Please select the one below that is the best fit for your research. If you are not sure, read the appropriate sections before making your selection.

☐ Life sciences ☐ Behavioural & social sciences ☒ Ecological, evolutionary & environmental sciences

For a reference copy of the document with all sections, see nature.com/documents/nr-reporting-summary-flat.pdf

Ecological, evolutionary & environmental sciences study design

All studies must disclose on these points even when the disclosure is negative.

Study description	We sequenced the genomes of 10 dingoes and 2 New Guinea Singing Dogs, to study the origins and feralization process of the dingo. First, we made phylogenetic and demographic analyses to study the origins of dingo. Then we made selection analysis to study feralization process of the dingo.
Research sample	10 dingoes and 2 New Guinea Singing Dogs
Sampling strategy	10 dingoes and 2 NGSDs were sequenced for the current study. The samples of dingoes have a wide distribution across Australia, and the two NGSDs are from the NGSD Conservation Society stud book.
Data collection	We sequenced the genomes of 10 dingoes and 2 New Guinea Singing Dogs in Berry Genomics Corporation, and work by Xinhui Han.
Timing and spatial scale	We sequenced the genomes in April 2015 to July 2015
Data exclusions	No data were excluded from the analysis
Reproducibility	All dual-luciferase reporter assay were performed in at least three independent experiments with a minimum of three replicates.
Randomization	The samples of dingoes have a wide distribution across Australia, and only 2 NGSDs avaliable.
Blinding	anlaysis and experiments are blinding in the present study

Did the study involve field work? ☐ Yes ☒ No

Reporting for specific materials, systems and methods

We require information from authors about some types of materials, experimental systems and methods used in many studies. Here, indicate whether each material, system or method listed is relevant to your study. If you are not sure if a list item applies to your research, read the appropriate section before selecting a response.

Materials & experimental systems

n/a	Involved in the study	
☒	☐	Antibodies
☐	☒	Eukaryotic cell lines
☒	☐	Palaeontology
☐	☒	Animals and other organisms
☒	☐	Human research participants
☒	☐	Clinical data

Methods

n/a	Involved in the study	
☒	☐	ChIP-seq
☒	☐	Flow cytometry
☒	☐	MRI-based neuroimaging

Eukaryotic cell lines

Policy information about cell lines

Cell line source(s)	HEK293 (human embryonic kidney cell line, KCB 200408YJ) was bought from Conervation Genetics CAS Kunming Cell Bank. Daoy (human medulloblastoma cell line, HTB-186) and MDCK (Canis normal kidney cell line, CLL-34) were from ATCC Global Bioresource Center.
Authentication	All of the three used cell lines were authenticated by Concervation Genetics CAS Kunming Cell Bank.
Mycoplasma contamination	All of the three used cell lines were tested negative for mycoplasma contamination by PCR-based analysis using the following primers: forward: 5'- ACTCCTACGGGAGGCAGCAGTA-3' and reverse: 5'-TGCACCATCTGTCACTCTGTTAACCTC-3'.
Commonly misidentified lines (See ICLAC register)	None.

October 2018

Animals and other organisms

Policy information about studies involving animals; ARRIVE guidelines recommended for reporting animal research

Laboratory animals	None.
Wild animals	J. William O. Ballard of the University of New South Wales for providing dingo samples and Janice Koler-Matznick for providing NGSD samples. Australian Government Export permit number N39585 and University of New South Wales Ethic's Approval 16/77B to Professor Bill Ballard.
Field-collected samples	None.
Ethics oversight	University of New South Wales

Note that full information on the approval of the study protocol must also be provided in the manuscript.
