# Supplementary material for: Genomic regions under selection in the feralization of the dingoes
Source: Nat Commun. 2020 Feb 3;11:671. doi: 10.1038/s41467-020-14515-6 (PMC6997406; doi:10.1038/s41467-020-14515-6)
Supplement: Supplementary file 4 — Description of Additional Supplementary Files [file 41467_2020_14515_MOESM4_ESM.pdf]

Description of Additional Supplementary Files

File Name: Supplementary Data 1
Description: Sample information

File Name: Supplementary Data 2
Description: The results of D-statistics (outgroup is Dhole)

File Name: Supplementary Data 3
Description: Results of the first G-phocs analysis

File Name: Supplementary Data 4
Description: The result of the second G-phocs analysis

File Name: Supplementary Data 5
Description: 87 candidate regions for selection during feralization, identified by overlap of PBS and iHS analysis

File Name: Supplementary Data 6
Description: Gene ontology analysis of the 50 feralization gene candidates
