# Supplementary material for: Genomic regions under selection in the feralization of the dingoes
Source: Nat Commun. 2020 Feb 3;11:671. doi: 10.1038/s41467-020-14515-6 (PMC6997406; doi:10.1038/s41467-020-14515-6)
Supplement: Supplementary file 5 — Supplementary Data 1 [file 41467_2020_14515_MOESM5_ESM.pdf]

Supplementary Date 1. Sample information.

Source	Internal ID	Abbreviation	Population Name	Location	Depth（X）	Population structure and phylogenetic analysis	Dtest and outgroup_f3 analysis	Demographic histories analysis	Mitochondrial genome analysis	selection analysis	haplotype analysis
4	Dingo	Dingo/D00	Dingo	Australia	6.79827	Yes	Yes	Yes	Yes	Yes	Yes
in this research	FAMBDIN00001	BDIN01/D01	Dingo	Australia	20.4141	Yes	Yes	Yes	Yes	Yes	Yes
in this research	FAMBDIN00002	BDIN02/D02	Dingo	Australia	12.5689	Yes	Yes	Yes	Yes	Yes	Yes
in this research	FAMBDIN00003	BDIN03/D03	Dingo	Australia	18.0196	Yes	Yes	Yes	Yes	Yes	Yes
in this research	FAMBDIN00004	BDIN04/D04	Dingo	Australia	17.9643	Yes	Yes	Yes	Yes	Yes	Yes
in this research	FAMBDIN00005	BDIN05/D05	Dingo	Australia	10.5603	Yes	Yes	Yes	Yes	Yes	Yes
in this research	FAMBDIN00006	BDIN06/D06	Dingo	Australia	17.5542	Yes	Yes	Yes	Yes	Yes	Yes
in this research	FAMBDIN00007	BDIN07/D07	Dingo	Australia	8.48633	Yes	Yes	Yes	Yes	Yes	Yes
in this research	FAMBDIN00008	BDIN08/D08	Dingo	Australia	15.4932	Yes	Yes	Yes	Yes	Yes	Yes
in this research	FAMBDIN00009	BDIN09/D09	Dingo	Australia	13.2343	Yes	Yes	Yes	Yes	Yes	Yes
in this research	FAMBDIN00010	BDIN10/D10	Dingo	Australia	19.6043	Yes	Yes	Yes	Yes	Yes	Yes
in this research	FAMBNGS00001	BNGS01	New Guinea Singing Dog	Papua New Guinea	11.5436	Yes	Yes	No	Yes	No	Yes
in this research	FAMBNGS00002	BNGS02	New Guinea Singing Dog	Papua New Guinea	11.5095	Yes	Yes	No	Yes	No	Yes
5	IN18	IN18	Indonesian indigenous dog	Oceania	4.94	Yes	Yes	Yes	Yes	No	No
5	IN23	IN23	Indonesian indigenous dog	Oceania	6.95	Yes	Yes	Yes	Yes	No	No
5	IN29	IN29	Indonesian indigenous dog	Oceania	5.74	Yes	Yes	Yes	Yes	No	No
1	FAMICHN00002	ICHN02	Chinese indigenous dog	China	10.9679	Yes	Yes	Yes	No	Yes	Yes
1	FAMICHN00010	ICHN10	Chinese indigenous dog	China	16.8591	Yes	Yes	Yes	No	Yes	Yes
1	FAMICHN00011	ICHN11	Chinese indigenous dog	China	16.2655	Yes	Yes	Yes	No	Yes	Yes
1	FAMICHN00021	ICHN21	Chinese indigenous dog	China	15.1386	Yes	Yes	Yes	No	Yes	Yes

1	FAMICHN00023	ICHN23	Chinese indigenous dog	China	15.8409	Yes	Yes	Yes	No	Yes	Yes
1	FAMICHN00025	ICHN25	Chinese indigenous dog	China	8.48146	Yes	Yes	Yes	No	Yes	Yes
5	TW04	TW04	Taiwan village dog	Taiwan China	14.94	Yes	Yes	No	No	No	No
6	YJ1	YJ1	Chinese indigenous dog	China	16.8933	Yes	Yes	Yes	No	Yes	Yes
6	YJ2	YJ2	Chinese indigenous dog	China	15.1174	Yes	Yes	Yes	No	Yes	Yes
6	YJ3	YJ3	Chinese indigenous dog	China	14.7926	Yes	Yes	Yes	No	Yes	Yes
6	YJ4	YJ4	Chinese indigenous dog	China	15.0133	Yes	Yes	Yes	No	Yes	Yes
6	YJ5	YJ5	Chinese indigenous dog	China	14.4965	Yes	Yes	Yes	No	Yes	Yes
6	YJ6	YJ6	Chinese indigenous dog	China	15.3986	Yes	Yes	Yes	No	Yes	Yes
6	YJ7	YJ7	Chinese indigenous dog	China	15.2466	Yes	Yes	Yes	No	Yes	Yes
6	YJ8	YJ8	Chinese indigenous dog	China	15.6179	Yes	Yes	Yes	No	Yes	Yes
6	YJ9	YJ9	Chinese indigenous dog	China	15.613	Yes	Yes	Yes	No	Yes	Yes
6	YJ10	YJ10	Chinese indigenous dog	China	16.2995	Yes	Yes	Yes	No	Yes	Yes
1	FAMIVNM00001	IVNM01	Vietnamese indigenous dog	Vietnam	16.5691	Yes	Yes	No	No	Yes	Yes
1	FAMIVNM00002	IVNM02	Vietnamese indigenous dog	Vietnam	17.3061	Yes	Yes	No	No	Yes	Yes
1	FAMIVNM00003	IVNM03	Vietnamese indigenous dog	Vietnam	14.5377	Yes	Yes	No	No	Yes	Yes
1	FAMIVNM00004	IVNM04	Vietnamese indigenous dog	Vietnam	18.495	Yes	Yes	No	No	Yes	Yes
1	FAMIVNM00005	IVNM05	Vietnamese indigenous dog	Vietnam	17.9326	Yes	Yes	No	No	Yes	Yes
5	VN21	VN21	Vietnamese indigenous dog	Vietnam	9.04	Yes	Yes	No	No	No	Yes
5	VN37	VN37	Vietnamese indigenous dog	Vietnam	6.77	Yes	Yes	No	No	No	Yes
5	VN4	VN4	Vietnamese indigenous dog	Vietnam	9.1	Yes	Yes	No	No	No	Yes
5	VN42	VN42	Vietnamese indigenous dog	Vietnam	9.08	Yes	Yes	No	No	No	Yes
5	VN59	VN59	Vietnamese indigenous dog	Vietnam	8.64	Yes	Yes	No	No	No	Yes
5	VN76	VN76	Vietnamese indigenous dog	Vietnam	6.11	Yes	Yes	No	No	No	Yes
1	FAMICHN00001	ICHN01	Chinese indigenous dog	China	13.5019	Yes	Yes	No	No	No	No
1	FAMICHN00003	ICHN03	Chinese indigenous dog	China	9.87969	Yes	Yes	No	No	No	No

1	FAMICHN00004	ICHN04	Chinese indigenous dog	China	16.3771	Yes	Yes	No	No	No	No
1	FAMICHN00005	ICHN05	Chinese indigenous dog	China	18.7594	Yes	Yes	No	No	No	No
1	FAMICHN00006	ICHN06	Chinese indigenous dog	China	15.0381	Yes	Yes	No	No	No	No
1	FAMICHN00007	ICHN07	Chinese indigenous dog	China	17.4137	Yes	Yes	No	No	No	No
1	FAMICHN00012	ICHN12	Chinese indigenous dog	China	17.7637	Yes	Yes	No	No	No	No
1	FAMICHN00014	ICHN14	Chinese indigenous dog	China	16.4811	Yes	Yes	No	No	No	No
1	FAMICHN00015	ICHN15	Chinese indigenous dog	China	15.9339	Yes	Yes	No	No	No	No
1	FAMICHN00016	ICHN16	Chinese indigenous dog	China	16.8995	Yes	Yes	No	No	No	No
1	FAMICHN00017	ICHN17	Chinese indigenous dog	China	12.0982	Yes	Yes	No	No	No	No
1	FAMICHN00019	ICHN19	Chinese indigenous dog	China	16.9206	Yes	Yes	No	No	No	No
1	FAMINGR00001	INGR01	Nigerian dog	Africa	16.6559	Yes	Yes	No	No	No	No
1	FAMINGR00002	INGR02	Nigerian dog	Africa	16.4833	Yes	Yes	No	No	No	No
1	FAMINGR00003	INGR03	Nigerian dog	Africa	14.7533	Yes	Yes	No	No	No	No
1	FAMINGR00004	INGR04	Nigerian dog	Africa	16.9414	Yes	Yes	No	No	No	No
5	PG115	PG115	Papua New Guinea village dog	Papua New Guinea	8.03	Yes	Yes	No	No	No	No
5	PG122	PG122	Papua New Guinea village dog	Papua New Guinea	5.75	Yes	Yes	No	No	No	No
5	PG84	PG84	Papua New Guinea village dog	Papua New Guinea	15	Yes	Yes	No	No	No	No
5	ID125	ID125	Indian village dog	India	12.66	Yes	Yes	No	No	No	No
5	ID137	ID137	Indian village dog	India	7.2	Yes	Yes	No	No	No	No
5	ID165	ID165	Indian village dog	India	9.47	Yes	Yes	No	No	No	No
5	ID168	ID168	Indian village dog	India	8.34	Yes	Yes	No	No	No	No
5	ID60	ID60	Indian village dog	India	13.02	Yes	Yes	No	No	No	No
5	ID91	ID91	Indian village dog	India	7.05	Yes	Yes	No	No	No	No
1	FAMBAFG00001	BAFG01	Afghan Hound	Afghanistan	16.2009	Yes	Yes	No	No	No	No
1	FAMBALM00001	BALM01	Alaskan Malamute	Alaska	15.1382	Yes	Yes	No	No	No	No
1	FAMBBEM00001	BBEM01	Belgian Malinois	Belgium	10.0814	Yes	Yes	No	No	No	No

1	FAMBCHI00001	BCHI01	Chihuahua	Mexico	16.6457	Yes	Yes	No	No	No	No
1	FAMBESL00001	BESL01	East Siberian laika	Russia	15.6143	Yes	Yes	No	No	No	No
1	FAMBFIL00001	BFIL01	Finnish Lapphund	Finland	14.5362	Yes	Yes	No	No	Yes	Yes
1	FAMBGAL00001	BGAL01	Galgo Español	Spain	13.5752	Yes	Yes	No	No	Yes	Yes
1	FAMBGNE00001	BGNE01	Grey Norwegian Elkhound	Norway	17.1074	Yes	Yes	No	No	Yes	Yes
1	FAMBGRD00001	BGRD01	Greenland Dog	Greenland	15.2095	Yes	Yes	No	No	Yes	No
1	FAMBGSD00001	BGSD01	German Shepherd Dog	Geremany	9.58868	Yes	Yes	No	No	Yes	Yes
1	FAMBJAM00001	BJAM01	Jämthund	Sweden	35.0732	Yes	Yes	No	No	Yes	Yes
1	FAMBLAH00001	BLAH01	Lapponian Herder	Finland	15.1344	Yes	Yes	No	No	Yes	Yes
1	FAMBMEN00001	BMEN01	Mexican naked (hairless)	Mexico	15.2126	Yes	Yes	No	No	Yes	Yes
1	FAMBPEN00001	BPEN01	Peruvian naked (hairless)	Peru	16.3519	Yes	Yes	No	No	Yes	Yes
1	FAMBSAM00001	BSAM01	samoyed	Russia	13.8903	Yes	Yes	No	No	No	No
1	FAMBSIH00001	BSIH01	Siberian Husky	Russia	12.9892	Yes	Yes	No	No	No	No
1	FAMBSLO00001	BSLO01	Sloughi	Morocco/Algeria/Tunisia/Libya	18.3573	Yes	Yes	No	No	No	No
1	FAMBSWL00001	BSWL01	Swedish Lapphund	Sweden	16.8476	Yes	Yes	No	No	Yes	Yes
1	FAMBTIM00001	BTIM01	Tibetan Mastiff	Tibet	11.0835	Yes	Yes	No	No	No	No
1	LUPZCHN00002	ZCHN02	Gray wolf	China	9.92547	Yes	Yes	No	No	Yes	Yes
1	LUPWCHN00003	WCHN03	Gray wolf	China	15.5493	Yes	Yes	No	No	Yes	Yes
1	LUPZCHN00005	ZCHN05	Gray wolf	China	8.61131	Yes	Yes	No	No	Yes	Yes
1	LUPZCHN00006	ZCHN06	Gray wolf	China	12.6777	Yes	Yes	No	No	Yes	Yes
1	LUPWCHN00008	WCHN08	Gray wolf	China	11.7294	Yes	Yes	No	No	Yes	Yes
1	LUPWCHN00009	WCHN09	Gray wolf	China	28.3691	Yes	Yes	No	No	Yes	Yes
1	LUPWCHN00010	WCHN10	Gray wolf	China	16.0579	Yes	Yes	No	No	Yes	Yes
1	LUPWCHN00013	WCHN13	Gray wolf	China	17.2867	Yes	Yes	No	No	Yes	Yes
1	LUPWCHN00001	WCHN01	Gray wolf	China	9.39317	Yes	Yes	No	No	Yes	Yes
1	LUPWRUS00001	WRUS01	Gray wolf	Russia	11.9743	Yes	Yes	No	No	Yes	Yes

1	LUPWRUS00002	WRUS02	Gray wolf	Russia	9.38865	Yes	Yes	No	No	Yes	Yes
1	LUPWRUS00003	WRUS03	Gray wolf	Russia	12.0891	Yes	Yes	No	No	Yes	Yes
2	Spanish wolf	SP01	Gray wolf	Spain	25.29	Yes	Yes	No	No	Yes	Yes
2	Italian wolf	IT01	Gray wolf	Italy	13.01	Yes	Yes	No	No	Yes	Yes
2	Portuguese wolf	PO01	Gray wolf	Portugal	26.1	Yes	Yes	No	No	Yes	Yes
2	Indian wolf	IN01	Gray wolf	India	26.03	Yes	Yes	No	No	Yes	Yes
2	Iranian wolf	IR01	Gray wolf	Iran	27.94	Yes	Yes	No	No	Yes	Yes
3	TI09	TI09	Gray_wolf	China	26.3449	Yes	Yes	No	No	Yes	Yes
3	TI32	TI32	Gray_wolf	China	25.8333	Yes	Yes	No	No	Yes	Yes
4	CHW	CHW	Gray_wolf	China	28.0405	Yes	Yes	No	No	Yes	Yes
4	CRW	CRW	Gray_wolf	Croatia	8.98117	Yes	Yes	No	No	Yes	Yes

[1]. Wang, G.D., et al., Out of southern East Asia: the natural history of domestic dogs across the world. Cell Res, 2016. 26(1): p. 21-33.

[2]. Fan, Z., et al., Worldwide patterns of genomic variation and admixture in gray wolves. Genome Research, 2016. 26(2): p. 163-173.

[3]. Zhang, W., et al., Hypoxia Adaptations in the Grey Wolf (Canis lupus chanco) from Qinghai-Tibet Plateau. PLOS Genetics,. 2014. 10(7).

[4]. Freedman, A.H., et al., Genome Sequencing Highlights the Dynamic Early History of Dogs. PLOS Genetics, 2014. 10(1).

[5]. Auton A, Li Y R, Kidd J, et al. Genetic recombination is targeted towards gene promoter regions in dogs[J]. PLoS genetics, 2013, 9(12): e1003984.

[6]. Gou, X., et al., Whole-genome sequencing of six dog breeds from continuous altitudes reveals adaptation to high-altitude hypoxia. Genome Research, 2014. 24(8): p. 1308-1315.
