# Supplementary material for: Genomic regions under selection in the feralization of the dingoes
Source: Nat Commun. 2020 Feb 3;11:671. doi: 10.1038/s41467-020-14515-6 (PMC6997406; doi:10.1038/s41467-020-14515-6)
Supplement: Supplementary file 6 — Supplementary Data 2 [file 41467_2020_14515_MOESM6_ESM.pdf]

Supplementary Date 2. The results of D-statistics (outgroup is Dhole).

Z value of D (Dhole, breed1; X, Y). Red indicates Z>3 and green indicates Z<-3.

Y \ X	D00	D01	D02	D03	D04	D05	D06	D07	D08	D09	D10
D00		-3.484	2.486	1.572	3.397	-5.731	-2.954	-3.181	0.476	3.855	3.176
D01	3.484		4.508	4.178	5.669	-2.184	-0.305	1.669	3.164	6.484	5.492
D02	-2.486	-4.508		-1.479	-0.028	-7.031	-6.21	-3.611	-2.036	0.495	0.078
D03	-1.572	-4.178	1.479		2.246	-7.344	-4.712	-3.08	-1.051	2.964	2.146
D04	-3.397	-5.669	0.028	-2.246		-9.615	-7.177	-4.853	-2.716	0.943	0.168
D05	5.731	2.184	7.031	7.344	9.615		1.616	4.039	5.768	10.758	9.727
D06	2.954	0.305	6.21	4.712	7.177	-1.616		1.519	3.175	7.438	6.597
D07	3.181	-1.669	3.611	3.08	4.853	-4.039	-1.519		1.887	5.253	4.457
D08	-0.476	-3.164	2.036	1.051	2.716	-5.768	-3.175	-1.887		3.472	2.674
D09	-3.855	-6.484	-0.495	-2.964	-0.943	-10.758	-7.438	-5.253	-3.472		-0.624
D10	-3.176	-5.492	-0.078	-2.146	-0.168	-9.727	-6.597	-4.457	-2.674	0.624	

Z value of D (Dhole, breed2; X, Y). Red indicates Z>3 and green indicates Z<-3.

Y \ X	D00	D01	D02	D03	D04	D05	D06	D07	D08	D09	D10
D00		-2.35	4.271	1.79	3.62	-4.437	-2.744	-2.154	0.823	3.997	3.267
D01	2.35		6.151	3.777	5.382	-2.173	-0.989	1.197	2.93	5.968	5.139
D02	-4.271	-6.151		-3.524	-1.204	-9.746	-7.829	-5.323	-3.373	-0.272	-1.362
D03	-1.79	-3.777	3.524		2.663	-6.359	-4.833	-2.911	-0.877	3.321	2.257
D04	-3.62	-5.382	1.204	-2.663		-8.911	-7.347	-4.699	-2.776	0.821	-0.308
D05	4.437	2.173	9.746	6.359	8.911		0.871	3.505	5.362	9.879	8.377
D06	2.744	0.989	7.829	4.833	7.347	-0.871		1.891	3.327	7.632	6.473
D07	2.154	-1.197	5.323	2.911	4.699	-3.505	-1.891		1.839	5.099	4.242

D08	-0.823	-2.93	3.373	0.877	2.776	-5.362	-3.327	-1.839		3.499	2.508
D09	-3.997	-5.968	0.272	-3.321	-0.821	-9.879	-7.632	-5.099	-3.499		-1.02
D10	-3.267	-5.139	1.362	-2.257	0.308	-8.377	-6.473	-4.242	-2.508	1.02	

Z value of D (Dhole,breed3;X,Y). Red indicates Z>3 and green indicates Z<-3.

Y \ X	D00	D01	D02	D03	D04	D05	D06	D07	D08	D09	D10
D00		-3.09	3.974	1.268	3.277	-4.875	-2.658	-2.276	0.603	3.877	3.931
D01	3.09		6.327	3.785	5.477	-1.895	-0.207	1.909	3.219	6.138	6.066
D02	-3.974	-6.327		-3.682	-1.121	-9.872	-7.349	-5.018	-3.383	-0.112	0.043
D03	-1.268	-3.785	3.682		2.766	-6.08	-4.097	-2.394	-0.591	3.454	3.714
D04	-3.277	-5.477	1.121	-2.766		-8.918	-6.989	-4.344	-2.706	0.912	1.059
D05	4.875	1.895	9.872	6.08	8.918		1.425	3.999	5.279	9.432	9.589
D06	2.658	0.207	7.349	4.097	6.989	-1.425		1.64	2.993	7.049	7.443
D07	2.276	-1.909	5.018	2.394	4.344	-3.999	-1.64		1.712	4.86	4.951
D08	-0.603	-3.219	3.383	0.591	2.706	-5.279	-2.993	-1.712		3.445	3.452
D09	-3.877	-6.138	0.112	-3.454	-0.912	-9.432	-7.049	-4.86	-3.445		0.145
D10	-3.931	-6.066	-0.043	-3.714	-1.059	-9.589	-7.443	-4.951	-3.452	-0.145	

Z value of D (Dhole,breed4;X,Y). Red indicates Z>3 and green indicates Z<-3.

Y \ X	D00	D01	D02	D03	D04	D05	D06	D07	D08	D09	D10
D00		-3.546	7.204	2.169	5.768	-4.532	-2.01	-3.383	0.808	5.832	4.97
D01	3.546		10.302	4.998	8.561	-1.024	0.753	2.04	3.64	9.095	7.784
D02	-7.204	-10.302		-5.428	-1.515	-12.973	-9.05	-9.101	-5.781	-1.345	-2.263
D03	-2.169	-4.998	5.428		4.079	-6.935	-4.245	-3.659	-1.275	4.159	3.299
D04	-5.768	-8.561	1.515	-4.079		-11.085	-8.473	-7.456	-4.634	0.087	-0.697
D05	4.532	1.024	12.973	6.935	11.085		1.588	2.974	5.438	11.897	10.409

D06	2.01	-0.753	9.05	4.245	8.473	-1.588		0.756	2.589	8.081	7.126
D07	3.383	-2.04	9.101	3.659	7.456	-2.974	-0.756		2.171	7.527	6.605
D08	-0.808	-3.64	5.781	1.275	4.634	-5.438	-2.589	-2.171		5.277	4.101
D09	-5.832	-9.095	1.345	-4.159	-0.087	-11.897	-8.081	-7.527	-5.277		-0.771
D10	-4.97	-7.784	2.263	-3.299	0.697	-10.409	-7.126	-6.605	-4.101	0.771	

Z value of D (Dhole,breed5;X,Y). Red indicates Z>3 and green indicates Z<-3.

Y \ X	D00	D01	D02	D03	D04	D05	D06	D07	D08	D09	D10
D00		-2.343	5.286	2.268	3.859	-4.5	-1.769	-3.293	1.512	4.465	4.124
D01	2.343		6.525	3.778	5.122	-2.224	-0.213	0.354	3.259	5.914	5.397
D02	-5.286	-6.525		-4.424	-2.214	-11.295	-7.003	-6.624	-3.62	-1.034	-1.168
D03	-2.268	-3.778	4.424		2.509	-6.802	-3.837	-3.805	-0.608	3.221	2.853
D04	-3.859	-5.122	2.214	-2.509		-9.171	-5.941	-5.264	-2.372	0.889	0.654
D05	4.5	2.224	11.295	6.802	9.171		1.677	2.718	5.942	10.27	9.713
D06	1.769	0.213	7.003	3.837	5.941	-1.677		0.447	2.862	6.252	6.001
D07	3.293	-0.354	6.624	3.805	5.264	-2.718	-0.447		2.964	5.871	5.442
D08	-1.512	-3.259	3.62	0.608	2.372	-5.942	-2.862	-2.964		3.097	2.783
D09	-4.465	-5.914	1.034	-3.221	-0.889	-10.27	-6.252	-5.871	-3.097		-0.154
D10	-4.124	-5.397	1.168	-2.853	-0.654	-9.713	-6.001	-5.442	-2.783	0.154	

Z value of D (Dhole,breed6;X,Y). Red indicates Z>3 and green indicates Z<-3.

Y \ X	D00	D01	D02	D03	D04	D05	D06	D07	D08	D09	D10
D00		-3.224	2.566	1.565	2.701	-5.147	-3.216	-3.478	0.783	3.515	3.515
D01	3.224		4.575	4.066	4.807	-1.812	-0.939	1.566	3.404	6.001	5.662
D02	-2.566	-4.575		-1.547	-0.268	-7.119	-6.842	-3.828	-1.915	0.495	0.653
D03	-1.565	-4.066	1.547		1.579	-7.046	-5.098	-3.122	-0.801	2.74	2.823

D04	-2.701	-4.807	0.268	-1.579		-8.269	-6.361	-4.141	-1.964	1.001	1.163
D05	5.147	1.812	7.119	7.046	8.269		0.448	3.52	5.943	10.006	9.907
D06	3.216	0.939	6.842	5.098	6.361	-0.448		1.979	3.868	7.213	7.409
D07	3.478	-1.566	3.828	3.122	4.141	-3.52	-1.979		2.289	5.008	4.917
D08	-0.783	-3.404	1.915	0.801	1.964	-5.943	-3.868	-2.289		2.984	2.981
D09	-3.515	-6.001	-0.495	-2.74	-1.001	-10.006	-7.213	-5.008	-2.984		0.202
D10	-3.515	-5.662	-0.653	-2.823	-1.163	-9.907	-7.409	-4.917	-2.981	-0.202	

Z value of D (Dhole,breed7;X,Y). Red indicates Z>3 and green indicates Z<-3.

Y \ X	D00	D01	D02	D03	D04	D05	D06	D07	D08	D09	D10
D00		-2.929	4.641	2.121	4.106	-4.61	-2.29	-2.943	1.301	4.273	3.739
D01	2.929		6.597	4.083	5.852	-1.772	-0.156	1.128	3.536	6.295	5.565
D02	-4.641	-6.597		-3.553	-0.852	-10.983	-7.812	-6.003	-3.499	-0.419	-1.279
D03	-2.121	-4.083	3.553		3.025	-7.053	-4.735	-3.566	-0.801	3.131	2.536
D04	-4.106	-5.852	0.852	-3.025		-9.85	-7.885	-5.515	-2.965	0.343	-0.47
D05	4.61	1.772	10.983	7.053	9.85		1.459	3.031	5.97	10.73	9.391
D06	2.29	0.156	7.812	4.735	7.885	-1.459		0.976	3.293	7.529	7.157
D07	2.943	-1.128	6.003	3.566	5.515	-3.031	-0.976		2.715	5.568	5.111
D08	-1.301	-3.536	3.499	0.801	2.965	-5.97	-3.293	-2.715		3.331	2.658
D09	-4.273	-6.295	0.419	-3.131	-0.343	-10.73	-7.529	-5.568	-3.331		-0.751
D10	-3.739	-5.565	1.279	-2.536	0.47	-9.391	-7.157	-5.111	-2.658	0.751	

Z value of D (Dhole,breed8;X,Y). Red indicates Z>3 and green indicates Z<-3.

Y \ X	D00	D01	D02	D03	D04	D05	D06	D07	D08	D09	D10
D00		-2.779	7.45	2.636	5.903	-5.06	-1.358	-1.853	0.507	6.224	5.824
D01	2.779		8.856	4.634	7.787	-2.095	0.749	1.82	2.879	7.926	7.687

Y \ X	D00	D01	D02	D03	D04	D05	D06	D07	D08	D09	D10
D02	-7.45	-8.856		-4.757	-0.961	-12.103	-7.081	-8.04	-6.083	-0.804	-0.832
D03	-2.636	-4.634	4.757		3.747	-7.246	-3.599	-3.366	-2.078	3.885	3.833
D04	-5.903	-7.787	0.961	-3.747		-10.937	-7.014	-6.853	-5.193	0.103	0.135
D05	5.06	2.095	12.103	7.246	10.937		2.485	4.092	5.296	11.04	11.108
D06	1.358	-0.749	7.081	3.599	7.014	-2.485		0.596	1.724	6.549	6.488
D07	1.853	-1.82	8.04	3.366	6.853	-4.092	-0.596		1.339	7.075	6.572
D08	-0.507	-2.879	6.083	2.078	5.193	-5.296	-1.724	-1.339		5.778	5.316
D09	-6.224	-7.926	0.804	-3.885	-0.103	-11.04	-6.549	-7.075	-5.778		0.029
D10	-5.824	-7.687	0.832	-3.833	-0.135	-11.108	-6.488	-6.572	-5.316	-0.029	

Z value of D (Dhole, breed9; X, Y). Red indicates Z>3 and green indicates Z<-3.

Y \ X	D00	D01	D02	D03	D04	D05	D06	D07	D08	D09	D10
D00		-2.323	2.264	1.718	4.297	-3.938	-2.324	-2.568	1.009	4.479	3.474
D01	2.323		3.567	3.441	5.735	-1.683	-0.768	0.917	2.864	5.997	4.947
D02	-2.264	-3.567		-1.239	1.058	-5.395	-5.039	-3.157	-1.554	1.211	0.571
D03	-1.718	-3.441	1.239		3.557	-5.846	-4.079	-2.891	-0.671	3.76	2.566
D04	-4.297	-5.735	-1.058	-3.557		-9.365	-7.221	-5.5	-3.363	0.311	-0.726
D05	3.938	1.683	5.395	5.846	9.365		0.604	2.673	4.807	9.602	8.502
D06	2.324	0.768	5.039	4.079	7.221	-0.604		1.369	3.106	7.099	6.061
D07	2.568	-0.917	3.157	2.891	5.5	-2.673	-1.369		2.186	5.657	4.581
D08	-1.009	-2.864	1.554	0.671	3.363	-4.807	-3.106	-2.186		3.723	2.705
D09	-4.479	-5.997	-1.211	-3.76	-0.311	-9.602	-7.099	-5.657	-3.723		-1.02
D10	-3.474	-4.947	-0.571	-2.566	0.726	-8.502	-6.061	-4.581	-2.705	1.02	

Z value of D (Dhole, breed10; X, Y). Red indicates Z>3 and green indicates Z<-3.

Y \ X	D00	D01	D02	D03	D04	D05	D06	D07	D08	D09	D10

D00		-2.51	4.58	2.437	4.013	-4.129	-1.895	-2.413	1.518	4.333	3.665
D01	2.51		6.205	4.327	5.757	-1.848	0.051	1.292	3.579	6.22	5.336
D02	-4.58	-6.205		-3.139	-0.876	-10.123	-7.805	-5.594	-3.183	-0.272	-1.161
D03	-2.437	-4.327	3.139		2.59	-7.115	-4.595	-3.505	-0.9	2.956	2.116
D04	-4.013	-5.757	0.876	-2.59		-9.692	-7.391	-5.05	-2.714	0.486	-0.388
D05	4.129	1.848	10.123	7.115	9.692		1.709	3.178	6.154	10.499	9.181
D06	1.895	-0.051	7.805	4.595	7.391	-1.709		0.894	3.185	7.301	6.592
D07	2.413	-1.292	5.594	3.505	5.05	-3.178	-0.894		2.577	5.355	4.648
D08	-1.518	-3.579	3.183	0.9	2.714	-6.154	-3.185	-2.577		3.092	2.384
D09	-4.333	-6.22	0.272	-2.956	-0.486	-10.499	-7.301	-5.355	-3.092		-0.839
D10	-3.665	-5.336	1.161	-2.116	0.388	-9.181	-6.592	-4.648	-2.384	0.839	

Z value of D (Dhole,breed11;X,Y). Red indicates Z>3 and green indicates Z<-3.

Y \ X	D00	D01	D02	D03	D04	D05	D06	D07	D08	D09	D10
D00		-2.541	4.33	1.9	3.664	-5.119	-2.412	-3.434	1.567	4.184	3.755
D01	2.541		5.82	3.765	5.154	-2.399	-0.311	0.819	3.731	6	5.313
D02	-4.33	-5.82		-3.242	-1.316	-10.289	-7.404	-5.881	-2.855	-0.214	-0.914
D03	-1.9	-3.765	3.242		2.387	-7.071	-4.46	-3.645	-0.284	3.146	2.609
D04	-3.664	-5.154	1.316	-2.387		-9.615	-6.816	-5.317	-2.173	0.893	0.306
D05	5.119	2.399	10.289	7.071	9.615		1.855	3.579	6.603	10.033	9.576
D06	2.412	0.311	7.404	4.46	6.816	-1.855		0.951	3.651	7.328	6.751
D07	3.434	-0.819	5.881	3.645	5.317	-3.579	-0.951		3.255	5.891	5.256
D08	-1.567	-3.731	2.855	0.284	2.173	-6.603	-3.651	-3.255		2.794	2.261
D09	-4.184	-6	0.214	-3.146	-0.893	-10.033	-7.328	-5.891	-2.794		-0.566
D10	-3.755	-5.313	0.914	-2.609	-0.306	-9.576	-6.751	-5.256	-2.261	0.566	

Z value of *D*(Andean_Fox,breed1;X,Y). Red indicates Z>3 and green indicates Z<-3.

Y \ X	D00	D01	D02	D03	D04	D05	D06	D07	D08	D09	D10
D00		-3.744	3.475	1.422	3.406	-5.687	-3.329	-3.714	0.235	3.725	3.382
D01	3.744		5.969	4.392	6.01	-1.97	-0.506	1.552	3.188	6.533	6.03
D02	-3.475	-5.969		-2.764	-0.593	-8.973	-7.664	-5.053	-3.005	-0.027	-0.444
D03	-1.422	-4.392	2.764		2.715	-7.04	-4.99	-3.324	-1.102	3.178	2.71
D04	-3.406	-6.01	0.593	-2.715		-9.536	-7.59	-5.108	-2.948	0.689	0.143
D05	5.687	1.97	8.973	7.04	9.536		1.22	3.815	5.348	10.315	9.491
D06	3.329	0.506	7.664	4.99	7.59	-1.22		1.629	3.324	7.687	7.579
D07	3.714	-1.552	5.053	3.324	5.108	-3.815	-1.629		1.974	5.385	4.997
D08	-0.235	-3.188	3.005	1.102	2.948	-5.348	-3.324	-1.974		3.539	2.997
D09	-3.725	-6.533	0.027	-3.178	-0.689	-10.315	-7.687	-5.385	-3.539		-0.479
D10	-3.382	-6.03	0.444	-2.71	-0.143	-9.491	-7.579	-4.997	-2.997	0.479	

Z value of *D*(Andean_Fox,breed2;X,Y). Red indicates Z>3 and green indicates Z<-3.

Y \ X	D00	D01	D02	D03	D04	D05	D06	D07	D08	D09	D10
D00		-3.083	4.123	1.58	3.572	-4.903	-3.151	-3.324	0.452	3.741	3.372
D01	3.083		6.447	4.239	5.868	-1.916	-0.862	1.247	3.073	6.216	5.725
D02	-4.123	-6.447		-3.774	-1.051	-9.936	-8.308	-5.651	-3.482	-0.456	-1.219
D03	-1.58	-4.239	3.774		3.043	-6.622	-5.11	-3.338	-1.012	3.315	2.764
D04	-3.572	-5.868	1.051	-3.043		-9.295	-7.757	-5.093	-3.02	0.471	-0.249
D05	4.903	1.916	9.936	6.622	9.295		0.795	3.36	5.166	10.036	8.824
D06	3.151	0.862	8.308	5.11	7.757	-0.795		1.76	3.399	7.75	7.4
D07	3.324	-1.247	5.651	3.338	5.093	-3.36	-1.76		2.019	5.271	4.861
D08	-0.452	-3.073	3.482	1.012	3.02	-5.166	-3.399	-2.019		3.476	2.854
D09	-3.741	-6.216	0.456	-3.315	-0.471	-10.036	-7.75	-5.271	-3.476		-0.672

Y \ X	D00	D01	D02	D03	D04	D05	D06	D07	D08	D09	D10
D10	-3.372	-5.725	1.219	-2.764	0.249	-8.824	-7.4	-4.861	-2.854	0.672	

Z value of D(Andean_Fox, breed3; X, Y). Red indicates $Z>3$ and green indicates $Z<-3$.

Y \ X	D00	D01	D02	D03	D04	D05	D06	D07	D08	D09	D10
D00		-3.515	4.147	1.303	3.337	-5.205	-3.053	-3.546	0.209	3.745	3.67
D01	3.515		6.719	4.205	5.868	-1.812	-0.398	1.55	3.11	6.335	6.202
D02	-4.147	-6.719		-4.091	-1.293	-10.277	-8.079	-5.794	-3.722	-0.499	-0.787
D03	-1.303	-4.205	4.091		2.995	-6.568	-4.634	-3.184	-0.991	3.444	3.506
D04	-3.337	-5.868	1.293	-2.995		-9.139	-7.318	-4.938	-3.042	0.633	0.471
D05	5.205	1.812	10.277	6.568	9.139		1.155	3.605	5	9.861	9.496
D06	3.053	0.398	8.079	4.634	7.318	-1.155		1.506	3.07	7.453	7.752
D07	3.546	-1.55	5.794	3.184	4.938	-3.605	-1.506		1.915	5.316	5.286
D08	-0.209	-3.11	3.722	0.991	3.042	-5	-3.07	-1.915		3.587	3.391
D09	-3.745	-6.335	0.499	-3.444	-0.633	-9.861	-7.453	-5.316	-3.587		-0.171
D10	-3.67	-6.202	0.787	-3.506	-0.471	-9.496	-7.752	-5.286	-3.391	0.171	

Z value of D(Andean_Fox, breed4; X, Y). Red indicates $Z>3$ and green indicates $Z<-3$.

Y \ X	D00	D01	D02	D03	D04	D05	D06	D07	D08	D09	D10
D00		-3.695	6.082	1.742	4.785	-5.194	-3.017	-3.99	0.444	5.104	4.514
D01	3.695		9.234	4.854	7.84	-1.452	-0.072	1.698	3.465	8.383	7.583
D02	-6.082	-9.234		-5.097	-1.602	-11.859	-8.983	-8.278	-5.086	-0.918	-1.873
D03	-1.742	-4.854	5.097		3.685	-6.875	-4.809	-3.723	-1.149	3.952	3.364
D04	-4.785	-7.84	1.602	-3.685		-10.472	-8.061	-6.816	-4.038	0.479	-0.289
D05	5.194	1.452	11.859	6.875	10.472		1.179	3.332	5.445	11.448	10
D06	3.017	0.072	8.983	4.809	8.061	-1.179		1.371	3.259	7.994	7.655
D07	3.99	-1.698	8.278	3.723	6.816	-3.332	-1.371		2.18	7.106	6.559

D08	-0.444	-3.465	5.086	1.149	4.038	-5.445	-3.259	-2.18		4.867	3.852
D09	-5.104	-8.383	0.918	-3.952	-0.479	-11.448	-7.994	-7.106	-4.867		-0.739
D10	-4.514	-7.583	1.873	-3.364	0.289	-10	-7.655	-6.559	-3.852	0.739	

Z value of *D*(Andean_Fox, breed5; X, Y). Red indicates Z>3 and green indicates Z<-3.

Y \ X	D00	D01	D02	D03	D04	D05	D06	D07	D08	D09	D10
D00		-3.028	4.725	1.81	3.579	-5.004	-2.682	-3.858	0.865	4.065	3.863
D01	3.028		6.63	4.043	5.506	-2.084	-0.57	0.736	3.199	6.164	5.792
D02	-4.725	-6.63		-4.372	-1.894	-10.865	-8.075	-6.462	-3.605	-0.93	-1.226
D03	-1.81	-4.043	4.372		2.815	-6.868	-4.599	-3.831	-0.767	3.371	3.158
D04	-3.579	-5.506	1.894	-2.815		-9.239	-6.95	-5.319	-2.629	0.737	0.465
D05	5.004	2.084	10.865	6.868	9.239		1.209	3.066	5.548	10.304	9.424
D06	2.682	0.57	8.075	4.599	6.95	-1.209		1.066	3.303	7.229	7.203
D07	3.858	-0.736	6.462	3.831	5.319	-3.066	-1.066		2.694	5.823	5.587
D08	-0.865	-3.199	3.605	0.767	2.629	-5.548	-3.303	-2.694		3.29	2.942
D09	-4.065	-6.164	0.93	-3.371	-0.737	-10.304	-7.229	-5.823	-3.29		-0.232
D10	-3.863	-5.792	1.226	-3.158	-0.465	-9.424	-7.203	-5.587	-2.942	0.232	

Z value of *D*(Andean_Fox, breed6; X, Y). Red indicates Z>3 and green indicates Z<-3.

Y \ X	D00	D01	D02	D03	D04	D05	D06	D07	D08	D09	D10
D00		-3.391	3.374	1.552	3.017	-5.181	-3.43	-3.893	0.485	3.569	3.466
D01	3.391		5.643	4.239	5.324	-1.792	-1.003	1.31	3.218	6.063	5.803
D02	-3.374	-5.643		-2.644	-0.747	-8.605	-7.89	-4.983	-2.838	0.02	-0.095
D03	-1.552	-4.239	2.644		2.187	-6.914	-5.367	-3.426	-1.03	2.921	2.888
D04	-3.017	-5.324	0.747	-2.187		-8.673	-7.313	-4.678	-2.515	0.822	0.723
D05	5.181	1.792	8.605	6.914	8.673		0.45	3.298	5.39	9.974	9.322

D06	3.43	1.003	7.89	5.367	7.313	-0.45		1.885	3.769	7.744	7.991
D07	3.893	-1.31	4.983	3.426	4.678	-3.298	-1.885		2.26	5.244	5.096
D08	-0.485	-3.218	2.838	1.03	2.515	-5.39	-3.769	-2.26		3.253	3.024
D09	-3.569	-6.063	-0.02	-2.921	-0.822	-9.974	-7.744	-5.244	-3.253		-0.124
D10	-3.466	-5.803	0.095	-2.888	-0.723	-9.322	-7.991	-5.096	-3.024	0.124	

Z value of *D*(Andean_Fox,breed7;X,Y). Red indicates Z>3 and green indicates Z<-3.

Y \ X	D00	D01	D02	D03	D04	D05	D06	D07	D08	D09	D10
D00		-3.34	4.455	1.664	3.831	-5.008	-2.982	-3.738	0.651	3.899	3.611
D01	3.34		6.871	4.26	6.15	-1.909	-0.586	1.066	3.299	6.4	5.97
D02	-4.455	-6.871		-4.041	-1.13	-10.686	-8.686	-6.201	-3.727	-0.709	-1.413
D03	-1.664	-4.26	4.041		3.265	-6.847	-5.013	-3.588	-0.926	3.23	2.964
D04	-3.831	-6.15	1.13	-3.265		-9.637	-8.247	-5.534	-3.15	0.243	-0.333
D05	5.008	1.909	10.686	6.847	9.637		1.078	3.145	5.451	10.429	9.257
D06	2.982	0.586	8.686	5.013	8.247	-1.078		1.341	3.44	7.815	7.841
D07	3.738	-1.066	6.201	3.588	5.534	-3.145	-1.341		2.433	5.556	5.321
D08	-0.651	-3.299	3.727	0.926	3.15	-5.451	-3.44	-2.433		3.453	2.953
D09	-3.899	-6.4	0.709	-3.23	-0.243	-10.429	-7.815	-5.556	-3.453		-0.527
D10	-3.611	-5.97	1.413	-2.964	0.333	-9.257	-7.841	-5.321	-2.953	0.527	

Z value of *D*(Andean_Fox,breed8;X,Y). Red indicates Z>3 and green indicates Z<-3.

Y \ X	D00	D01	D02	D03	D04	D05	D06	D07	D08	D09	D10
D00		-3.217	6.103	1.969	4.848	-5.392	-2.795	-3.289	0.321	5.15	4.91
D01	3.217		8.33	4.531	7.371	-2.111	-0.236	1.411	2.968	7.686	7.411
D02	-6.103	-8.33		-4.664	-1.263	-11.362	-8.249	-7.831	-5.056	-0.759	-1.176
D03	-1.969	-4.531	4.664		3.442	-7.015	-4.684	-3.681	-1.506	3.735	3.609

D04	-4.848	-7.371	1.263	-3.442		-10.162	-7.653	-6.64	-4.245	0.363	0.098
D05	5.392	2.111	11.362	7.015	10.162		1.594	3.865	5.262	10.763	10.225
D06	2.795	0.236	8.249	4.684	7.653	-1.594		1.326	2.986	7.43	7.513
D07	3.289	-1.411	7.831	3.681	6.64	-3.865	-1.326		1.861	6.949	6.659
D08	-0.321	-2.968	5.056	1.506	4.245	-5.262	-2.986	-1.861		4.785	4.363
D09	-5.15	-7.686	0.759	-3.735	-0.363	-10.763	-7.43	-6.949	-4.785		-0.262
D10	-4.91	-7.411	1.176	-3.609	-0.098	-10.225	-7.513	-6.659	-4.363	0.262	

Z value of *D*(Andean_Fox, breed9; X, Y). Red indicates Z>3 and green indicates Z<-3.

Y \ X	D00	D01	D02	D03	D04	D05	D06	D07	D08	D09	D10
D00		-3.106	3.21	1.464	3.836	-4.718	-3.004	-3.741	0.54	3.998	3.463
D01	3.106		5.313	3.964	5.991	-1.786	-0.833	0.94	3.06	6.273	5.662
D02	-3.21	-5.313		-2.59	0.288	-7.885	-7.1	-4.822	-2.67	0.477	-0.073
D03	-1.464	-3.964	2.59		3.452	-6.293	-4.713	-3.386	-0.849	3.572	2.959
D04	-3.836	-5.991	-0.288	-3.452		-9.34	-7.843	-5.562	-3.245	0.281	-0.434
D05	4.718	1.786	7.885	6.293	9.34		0.678	2.899	4.956	9.784	8.826
D06	3.004	0.833	7.1	4.713	7.843	-0.678		1.472	3.366	7.743	7.33
D07	3.741	-0.94	4.822	3.386	5.562	-2.899	-1.472		2.333	5.743	5.191
D08	-0.54	-3.06	2.67	0.849	3.245	-4.956	-3.366	-2.333		3.577	2.939
D09	-3.998	-6.273	-0.477	-3.572	-0.281	-9.784	-7.743	-5.743	-3.577		-0.683
D10	-3.463	-5.662	0.073	-2.959	0.434	-8.826	-7.33	-5.191	-2.939	0.683	

Z value of *D*(Andean_Fox, breed10; X, Y). Red indicates Z>3 and green indicates Z<-3.

Y \ X	D00	D01	D02	D03	D04	D05	D06	D07	D08	D09	D10
D00		-3.15	4.285	1.857	3.698	-4.788	-2.847	-3.493	0.766	3.976	3.541
D01	3.15		6.456	4.352	5.903	-1.834	-0.417	1.198	3.33	6.334	5.764

D02	-4.285	-6.456		-3.647	-0.956	-10.02	-8.508	-5.867	-3.398	-0.395	-1.178
D03	-1.857	-4.352	3.647		2.962	-6.898	-5.022	-3.647	-0.971	3.229	2.673
D04	-3.698	-5.903	0.956	-2.962		-9.428	-7.848	-5.243	-2.945	0.445	-0.262
D05	4.788	1.834	10.02	6.898	9.428		1.216	3.258	5.539	10.416	9.16
D06	2.847	0.417	8.508	5.022	7.848	-1.216		1.295	3.385	7.774	7.507
D07	3.493	-1.198	5.867	3.647	5.243	-3.258	-1.295		2.422	5.558	5.103
D08	-0.766	-3.33	3.398	0.971	2.945	-5.539	-3.385	-2.422		3.324	2.757
D09	-3.976	-6.334	0.395	-3.229	-0.445	-10.416	-7.774	-5.558	-3.324		-0.672
D10	-3.541	-5.764	1.178	-2.673	0.262	-9.16	-7.507	-5.103	-2.757	0.672	

Z value of *D*(Andean_Fox, breed11; X, Y). Red indicates Z>3 and green indicates Z<-3.

Y \ X	D00	D01	D02	D03	D04	D05	D06	D07	D08	D09	D10
D00		-3.24	4.238	1.64	3.518	-5.242	-2.863	-3.959	0.849	3.946	3.525
D01	3.24		6.507	4.238	5.828	-2.113	-0.429	1.006	3.595	6.448	5.86
D02	-4.238	-6.507		-3.774	-1.416	-10.225	-8.165	-6.033	-3.308	-0.406	-1.323
D03	-1.64	-4.238	3.774		2.762	-6.874	-4.745	-3.705	-0.681	3.361	2.825
D04	-3.518	-5.828	1.416	-2.762		-9.304	-7.339	-5.352	-2.64	0.804	0.015
D05	5.242	2.113	10.225	6.874	9.304		1.424	3.431	5.821	10.027	9.254
D06	2.863	0.429	8.165	4.745	7.339	-1.424		1.172	3.495	7.639	7.35
D07	3.959	-1.006	6.033	3.705	5.352	-3.431	-1.172		2.759	5.778	5.312
D08	-0.849	-3.595	3.308	0.681	2.64	-5.821	-3.495	-2.759		3.23	2.625
D09	-3.946	-6.448	0.406	-3.361	-0.804	-10.027	-7.639	-5.778	-3.23		-0.745
D10	-3.525	-5.86	1.323	-2.825	-0.015	-9.254	-7.35	-5.312	-2.625	0.745	

*breed1= FAMBBEM00001, breed2= FAMBCHI00001, breed3= FAMBFIL00001, breed4= FAMBGAL00001, breed5= FAMBGNE00001, breed6= FAMBGSD00001, breed7= FAMBJAM00001, breed8= FAMBLAH00001, breed9= FAMBMEN00001, breed10= FAMBPEN00001, breed11= FAMBSWL00001
