# Supplementary material for: Genomic regions under selection in the feralization of the dingoes
Source: Nat Commun. 2020 Feb 3;11:671. doi: 10.1038/s41467-020-14515-6 (PMC6997406; doi:10.1038/s41467-020-14515-6)
Supplement: Supplementary file 7 — Supplementary Data 3 [file 41467_2020_14515_MOESM7_ESM.pdf]

Supplementary information, Data 3. Results of the first G-phocs analysis.

(SV dog, (IN DOG, DINGO))								
Parameter	$N_e^{dog\ ancenstry}$	$N_e^{SV\ dog}$	$N_e^{IN\ dog}$	N_e^{dingo}	$N_e^{IN\&dingo\ ancenstry}$	Tau2	Tau1	m_SV->IN
Estimated value	1.50E-03(CI: 1.45 E-03 – 1.57 E-03)	4.73E-04(CI: 2.83E-04 –6.46E-04)	5.10E-04(CI: 1.89E-04 –8.26E-04)	3.32E-05(CI: 1.81E-05 – 4.27E-05)	2.43E-04(CI:4.31E-06 – 6.93E-04)	1.08E-05(CI:7.02E-06 – 1.46E-05)	1.29E-05(CI:8.41E-06 – 1.65E-05)	1985.96
In real units	96153.85(CI: 90625 - 98125)	30320.51(CI:18121 – 41403)	32670.94(CI:12134 – 52929)	2128.21(CI:1160 – 2737)	15598.29(CI:276 – 44410)	8307.69(CI:5400 – 12300)	9923.08(CI:6469 – 12692)	0.60*

SV dog is Indigenous dog from southern China, and IN dog is Indonesian village dog.
* this value is the population size of migrants per generation
