# Supplementary material for: Genomic regions under selection in the feralization of the dingoes
Source: Nat Commun. 2020 Feb 3;11:671. doi: 10.1038/s41467-020-14515-6 (PMC6997406; doi:10.1038/s41467-020-14515-6)
Supplement: Supplementary file 8 — Supplementary Data 4 [file 41467_2020_14515_MOESM8_ESM.pdf]

Supplementary information, Data 4. The result of the second G-phocs analysis.

(SV dog, (IN DOG, DINGO))								
Parameter	$N_e^{dog\ ancenstry}$	$N_e^{SV\ dog}$	$N_e^{IN\ dog}$	N_e^{dingo}	$N_e^{IN\&dingo\ ancenstry}$	Tau2	Tau1	m_SV->IN
Estimated value	1.52E-03(CI:1.42 – 1.61)	5.41E-04(CI:1.95E-04 – 9.72E-04	7.01E-04(CI:2.06E-04 – 1.41E-03)	3.35E-05(CI: 1.59E-05 – 4.98 E-05)	1.92E-04(CI:5.07E-06 – 4.70E-04)	1.02E-05(4.51E-06 – 1.55E-05)	1.26E-05(CI:5.42E-06 – 1.82E-05)	1992.47
In real units	97675.21(CI:91205 – 103205)	34698.72(CI:12525 – 62314)	44923.08(CI:13230 – 90205)	2147.44(CI:1019 – 3192)	12314.10(CI:325 – 30108)	7843.38(CI:3470 – 11900)	9666.67(CI:5420 – 14000)	0.82*

SV dog is Indigenous dog from southern China, and IN dog is Indonesian village dog.

*this value is the population size of migrants per generation.
