# Supplementary material for: Genomic regions under selection in the feralization of the dingoes
Source: Nat Commun. 2020 Feb 3;11:671. doi: 10.1038/s41467-020-14515-6 (PMC6997406; doi:10.1038/s41467-020-14515-6)
Supplement: Supplementary file 9 — Supplementary Data 5 [file 41467_2020_14515_MOESM9_ESM.pdf]

Supplementary Data 5. 87 candidate regions for selection during feralization, identified by overlap of PBS and iHS analysis

Chr	Start	End	PBS1 value	Proportion of sites above iHS threshold	Gene
chr1	3380001	3400001	0.24976331	0.416666667	*ZNF516*
chr1	26540001	26560001	0.18227655	0.5	
chr1	44760001	44780001	0.3493017	0.428571429	
chr1	84340001	84360001	0.17572208	0.384615385	
chr1	89060001	89080001	0.2016904	0.339622642	*CBWD2*
chr1	89080001	89100001	0.19374261	0.734693878	*CBWD2*
chr1	121880001	121900001	0.1452148	0.333333333	
chr2	5900001	5920001	0.21653451	1	*ENSCAFG00000003859*
chr2	51500001	51520001	0.14486967	0.363636364	*NLN*
chr2	54600001	54620001	0.27132863	0.70212766	*SMN*
chr2	58580001	58600001	0.15274268	0.343283582	
chr2	82460001	82480001	0.15670423	0.333333333	
chr3	69060001	69080001	0.15082702	0.352941176	*CLNK*
chr3	69900001	69920001	0.16590888	0.694444444	*STX18*
chr3	72540001	72560001	0.15853828	0.62962963	*PDS5A*
chr3	72700001	72720001	0.23687838	0.383333333	*UBE2K*
chr3	72720001	72740001	0.22007169	0.375	*UBE2K*
chr4	45740001	45760001	0.18594733	0.307692308	
chr4	61160001	61180001	0.15087172	0.310344828	*ARL15*
chr4	71360001	71380001	0.19619386	0.5	*NUP155, RF00415*
chr5	1720001	1740001	0.22716344	0.520833333	
chr5	1880001	1900001	0.16470817	0.397590361	
chr5	1900001	1920001	0.17125631	0.601941748	

chr5	24400001	24420001	0.14895443	0.421052632	*ACAT1, CUL5*
chr5	24440001	24460001	0.14599628	0.333333333	*ACAT1, CUL5*
chr5	39720001	39740001	0.18492095	0.333333333	*NCOR1*
chr6	23860001	23880001	0.16795761	0.459459459	*ENSCAFG00000017787, ENSCAFG00000023577*
chr6	23900001	23920001	0.23813311	0.890625	*ENSCAFG00000017787, ENSCAFG00000023577*
chr6	23960001	23980001	0.22971541	0.421686747	*ENSCAFG00000017787, ENSCAFG00000023577, ENSCAFG00000029739*
chr7	20140001	20160001	0.14887635	0.447761194	*FAM163A*
chr7	66280001	66300001	0.27994051	0.75	*MIB1*
chr8	24100001	24120001	0.1524322	0.363636364	*MDGA2*
chr8	47600001	47620001	0.16873642	0.506849315	
chr8	68600001	68620001	0.14485829	0.363636364	*SLC25A47, WARS*
chr9	17400001	17420001	0.24128744	1	
chr9	18120001	18140001	0.41999257	0.333333333	
chr9	49160001	49180001	0.21231142	0.301204819	*SEC16A, CCDC187*
chr10	7740001	7760001	0.1890376	0.388888889	
chr10	13800001	13820001	0.25772626	0.6	*TRHDE*
chr10	17020001	17040001	0.31213539	0.317460317	*TUBGCP6, SELENOO*
chr10	17040001	17060001	0.23049375	0.350877193	*ENSCAFG00000000697, TRABD, PANX2*
chr10	52260001	52280001	0.22360832	0.4375	
chr10	56560001	56580001	0.26154504	0.325396825	
chr10	57700001	57720001	0.19619589	0.363636364	
chr10	57720001	57740001	0.18644581	0.388888889	
chr11	41360001	41380001	0.17858935	0.463414634	
chr11	52380001	52400001	0.18381895	0.857142857	*TMEM8B, OR13J1*
chr11	57540001	57560001	0.16211405	0.580645161	

chr12	14820001	14840001	0.23459785	0.428571429	*SLC25A27*
chr13	25000001	25020001	0.18724275	0.452380952	
chr14	21500001	21520001	0.17060618	0.350877193	
chr15	60140001	60160001	0.2023676	0.525252525	
chr16	7300001	7320001	0.30768744	0.653846154	
chr16	7340001	7360001	0.32807274	0.461538462	*PRSS37, ENSCAFG00000003879*
chr16	7400001	7420001	0.27633819	0.5	*RF00026, TAS2R5*
chr17	560001	580001	0.31607615	0.336633663	
chr18	4940001	4960001	0.21015117	0.725806452	
chr18	4960001	4980001	0.19523387	0.848484848	
chr20	3960001	3980001	0.20362067	0.375	*ENSCAFG00000004411*
chr22	13280001	13300001	0.2445362	0.545454545	
chr22	13760001	13780001	0.15895738	0.333333333	
chr22	19980001	20000001	0.16223874	0.444444444	
chr22	27000001	27020001	0.31989736	0.363636364	*PIBF1*
chr22	27040001	27060001	0.27860691	0.6	*PIBF1*
chr22	59220001	59240001	0.14996748	0.358024691	*ARHGEF7*
chr23	16700001	16720001	0.1762338	0.333333333	*SLC4A7*
chr23	26980001	27000001	0.19689526	0.409090909	*ANKRD28*
chr23	45220001	45240001	0.17575541	0.328571429	*EIF2A*
chr25	19940001	19960001	0.14522631	0.565217391	*ENSCAFG00000025297*
chr25	20580001	20600001	0.16861347	0.590909091	*CLCN3*
chr25	29380001	29400001	0.16133545	0.368421053	
chr25	33020001	33040001	0.14774861	0.352941176	
chr26	24920001	24940001	0.19648726	0.454545455	*SLC5A1*
chr26	24940001	24960001	0.23197713	0.325	*SLC5A1*

chr27	29240001	29260001	0.14571238	0.609195402	
chr30	260001	280001	0.16884848	0.436363636	*ENSCAFG00000024251*
chr30	280001	300001	0.14926615	0.44	
chr30	300001	320001	0.14916176	0.413043478	
chr30	320001	340001	0.15141308	0.537037037	
chr30	340001	360001	0.17356658	0.935064935	
chr30	38640001	38660001	0.1613722	0.6	
chr31	21160001	21180001	0.15651709	0.304347826	*JAM2*
chr33	29480001	29500001	0.24649069	0.326086957	*UBXN7*
chr34	5560001	5580001	0.15607872	0.322580645	
chr36	7020001	7040001	0.18370027	0.6	
chr36	7040001	7060001	0.18299266	0.357142857	
chr37	20260001	20280001	0.15118421	0.6	
