# Supplementary material for: Genomic regions under selection in the feralization of the dingoes
Source: Nat Commun. 2020 Feb 3;11:671. doi: 10.1038/s41467-020-14515-6 (PMC6997406; doi:10.1038/s41467-020-14515-6)
Supplement: Supplementary file 10 — Supplementary Data 6 [file 41467_2020_14515_MOESM10_ESM.pdf]

**Supplementary Data 6.** Gene ontology analysis of the 50 feralization gene candidates

| GO.ID | GO Description | Gene Number | Gene | Category | Parent-Child TopGO P-Value | Xperm | pperm |
|---|---|---|---|---|---|---|---|
| GO:0072522 | purine-containing compound biosynthetic process | 3 | ACAT1, NCOR1, NUP155 | BP | 0.0016 | 0 | 0.000999001 |
| GO:0062012 | regulation of small molecule metabolic process | 4 | PIBF1, NLN, NCOR1, NUP155 | BP | 0.0024 | 0 | 0.000999001 |
| GO:0006109 | regulation of carbohydrate metabolic process | 3 | NLN, NCOR1, NUP155 | BP | 0.0025 | 0 | 0.000999001 |
| GO:1901293 | nucleoside phosphate biosynthetic process | 3 | ACAT1, NCOR1, NUP155 | BP | 0.0037 | 0 | 0.000999001 |
| GO:0060612 | adipose tissue development | 2 | ACAT1, ZNF516 | BP | 0.0039 | 0 | 0.000999001 |
| GO:0010256 | endomembrane system organization | 5 | CLCN3, STX18, ARHGEF7, NUP155, SEC16A | BP | 0.0046 | 0 | 0.000999001 |

| | | | | | | | |
|---|---|---|---|---|---|---|---|
| GO:0043603 | cellular amide metabolic process | 6 | TRHDE, NLN, WARS, EIF2A, NCOR1, NUP155 | BP | 0.005 | 0 | 0.000999001 |
| GO:0051188 | cofactor biosynthetic process | 3 | ACAT1, NCOR1, NUP155 | BP | 0.005 | 0 | 0.000999001 |
| GO:0006888 | ER to Golgi vesicle-mediated transport | 3 | ANKRD28, STX18, SEC16A | BP | 0.0052 | 0 | 0.000999001 |
| GO:0007030 | Golgi organization | 3 | STX18, ARHGEF7, SEC16A | BP | 0.0061 | 0 | 0.000999001 |
| GO:1901570 | fatty acid derivative biosynthetic process | 2 | PIBF1, ACAT1 | BP | 0.0072 | 0 | 0.000999001 |
| GO:0009266 | response to temperature stimulus | 3 | SLC25A27, NUP155, ZNF516 | BP | 0.0076 | 0 | 0.000999001 |
| GO:0072525 | pyridine-containing compound biosynthetic process | 2 | NCOR1, NUP155 | BP | 0.008 | 0 | 0.000999001 |
| GO:0046390 | ribose phosphate biosynthetic process | 3 | ACAT1, NCOR1, NUP155 | BP | 0.0106 | 0 | 0.000999001 |
| GO:0006903 | vesicle targeting | 2 | ANKRD28, SEC16A | BP | 0.0123 | 0 | 0.000999001 |

| | | | | | | | |
|---|---|---|---|---|---|---|---|
| GO:0007051 | spindle organization | 3 | PIBF1, TUBGCP6, NCOR1 | BP | 0.0125 | 0 | 0.000999001 |
| GO:0051225 | spindle assembly | 3 | PIBF1, TUBGCP6, NCOR1 | BP | 0.0195 | 0 | 0.000999001 |
| GO:1901568 | fatty acid derivative metabolic process | 2 | PIBF1, ACAT1 | BP | 0.0204 | 0 | 0.000999001 |
| GO:0060147 | regulation of posttranscriptional gene silencing | 2 | NCOR1, NUP155 | BP | 0.0233 | 0 | 0.000999001 |
| GO:0048208 | COPII vesicle coating | 2 | ANKRD28, SEC16A | BP | 0.0269 | 0 | 0.000999001 |
| GO:0007029 | endoplasmic reticulum organization | 2 | STX18, SEC16A | BP | 0.0109 | 3 | 0.003996004 |
| GO:0006082 | organic acid metabolic process | 5 | PIBF1, ACAT1, WARS, NCOR1, NUP155 | BP | 0.0144 | 3 | 0.003996004 |
| GO:0048193 | Golgi vesicle transport | 3 | ANKRD28, STX18, SEC16A | BP | 0.0191 | 3 | 0.003996004 |
| GO:0044283 | small molecule biosynthetic process | 5 | PIBF1, ACAT1, NLN, NCOR1, NUP155 | BP | 0.0013 | 4 | 0.004995005 |
| GO:0009409 | response to cold | 2 | SLC25A27, ZNF516 | BP | 0.0061 | 4 | 0.004995005 |
| GO:0051193 | regulation of cofactor metabolic process | 2 | NCOR1, NUP155 | BP | 0.0112 | 4 | 0.004995005 |

| | | | | | | | |
|---|---|---|---|---|---|---|---|
| GO:0043467 | regulation of generation of precursor metabolites and energy | 2 | NCOR1, NUP155 | BP | 0.0198 | 4 | 0.004995005 |
| GO:0006091 | generation of precursor metabolites and energy | 3 | ACAT1, NCOR1, NUP155 | BP | 0.0271 | 4 | 0.004995005 |
| GO:0060964 | regulation of gene silencing by miRNA | 2 | NCOR1, NUP155 | BP | 0.0333 | 4 | 0.004995005 |
| GO:0060968 | regulation of gene silencing | 2 | NCOR1, NUP155 | BP | 0.0349 | 4 | 0.004995005 |
| GO:0034404 | nucleobase-containing small molecule biosynthetic process | 2 | NCOR1, NUP155 | BP | 0.0363 | 8 | 0.008991009 |
| GO:0048839 | inner ear development | 2 | SLC25A27, SLC4A7 | BP | 0.0387 | 8 | 0.008991009 |
| GO:0030811 | regulation of nucleotide catabolic process | 2 | NCOR1, NUP155 | BP | 0.0045 | 9 | 0.00999001 |
| GO:0006110 | regulation of glycolytic process | 2 | NCOR1, NUP155 | BP | 0.0193 | 9 | 0.00999001 |

| | | | | | | | |
|---|---|---|---|---|---|---|---|
| GO:0043470 | regulation of carbohydrate catabolic process | 2 | NCOR1, NUP155 | BP | 0.0302 | 9 | 0.00999001 |
| GO:0060966 | regulation of gene silencing by RNA | 2 | NCOR1, NUP155 | BP | 0.0218 | 12 | 0.012987013 |
| GO:0016053 | organic acid biosynthetic process | 3 | PIBF1, NCOR1, NUP155 | BP | 0.0168 | 13 | 0.013986014 |
| GO:0051186 | cofactor metabolic process | 3 | ACAT1, NCOR1, NUP155 | BP | 0.0385 | 14 | 0.014985015 |
| GO:0030808 | regulation of nucleotide biosynthetic process | 2 | NCOR1, NUP155 | BP | 0.0138 | 16 | 0.016983017 |
| GO:0006140 | regulation of nucleotide metabolic process | 2 | NCOR1, NUP155 | BP | 0.0318 | 16 | 0.016983017 |
| GO:0072524 | pyridine-containing compound metabolic process | 2 | NCOR1, NUP155 | BP | 0.0302 | 18 | 0.018981019 |
| GO:0006811 | ion transport | 8 | CLCN3, SLC25A47, PANX2, SLC5A1, CUL5, SLC25A27, SLC4A7, NCOR1 | BP | 0.0306 | 18 | 0.018981019 |
| GO:0006900 | vesicle budding from membrane | 2 | ANKRD28, SEC16A | BP | 0.0216 | 19 | 0.01998002 |

| | | | | | | | |
|---|---|---|---|---|---|---|---|
| GO:0019538 | protein metabolic process | 13 | PIBF1, PRSS37, UBXN7, TRHDE, UBE2K, NLN, MIB1, WARS, CUL5, SELENOO, EIF2A, NCOR1, NUP155 | BP | 0.0224 | 20 | 0.020979021 |
| GO:0072521 | purine-containing compound metabolic process | 3 | ACAT1, NCOR1, NUP155 | BP | 0.038 | 30 | 0.030969031 |
| GO:0006464 | cellular protein modification process | 10 | PIBF1, UBXN7, UBE2K, MIB1, WARS, CUL5, SELENOO, EIF2A, NCOR1, NUP155 | BP | 0.038 | 36 | 0.036963037 |
| GO:0044281 | small molecule metabolic process | 6 | PIBF1, ACAT1, NLN, WARS, NCOR1, NUP155 | BP | 0.0407 | 44 | 0.044955045 |
| GO:1901564 | organonitrogen compound metabolic process | 14 | PIBF1, PRSS37, UBXN7, TRHDE, UBE2K, ACAT1, NLN, MIB1, WARS, CUL5, SELENOO, EIF2A, NCOR1, NUP155 | BP | 0.0259 | 47 | 0.047952048 |
| GO:0008233 | peptidase activity | 3 | PRSS37, TRHDE, NLN | MF | 0.0583 | 4 | 0.004995005 |
| GO:0015291 | secondary active transmembrane transporter activity | 3 | CLCN3, SLC5A1, SLC4A7 | MF | 0.2806 | 8 | 0.008991009 |
| GO:0033613 | activating transcription factor binding | 2 | NCOR1, ZNF516 | MF | 0.0455 | 12 | 0.012987013 |
| GO:0015370 | solute:sodium symporter activity | 2 | SLC5A1, SLC4A7 | MF | 0.1604 | 12 | 0.012987013 |
| GO:0005215 | transporter activity | 7 | CLCN3, SLC25A47, PANX2, SLC5A1, CUL5, SLC4A7, NUP155 | MF | 0.0045 | 20 | 0.020979021 |

| | | | | | | | |
|---|---|---|---|---|---|---|---|
| GO:0019787 | ubiquitin-like protein transferase activity | 3 | UBE2K, MIB1, CUL5 | MF | 0.0679 | 20 | 0.020979021 |
| GO:0044389 | ubiquitin-like protein ligase binding | 3 | UBXN7, UBE2K, CUL5 | MF | 0.0603 | 21 | 0.021978022 |
| GO:0015294 | solute:cation symporter activity | 2 | SLC5A1, SLC4A7 | MF | 0.1248 | 21 | 0.021978022 |
| GO:0008324 | cation transmembrane transporter activity | 5 | CLCN3, SLC25A47, SLC5A1, CUL5, SLC4A7 | MF | 0.2043 | 33 | 0.033966034 |
| GO:0015103 | inorganic anion transmembrane transporter activity | 2 | CLCN3, SLC4A7 | MF | 0.127 | 35 | 0.035964036 |
| GO:0035639 | purine ribonucleoside triphosphate binding | 6 | CLCN3, CBWD2, UBE2K, ARL15, WARS, SELENOO | MF | 0.058 | 36 | 0.036963037 |
| GO:0000139 | Golgi membrane | 4 | CLCN3, ANKRD28, STX18, SEC16A | CC | 0.0129 | 4 | 0.004995005 |
| GO:0005739 | mitochondrion | 6 | SLC25A47, ACAT1, TMEM8B, NLN, SELENOO, SLC25A27 | CC | 0.0543 | 7 | 0.007992008 |
| GO:0030658 | transport vesicle membrane | 2 | CLCN3, SEC16A | CC | 0.0372 | 25 | 0.025974026 |
| GO:0098858 | actin-based cell projection | 2 | UBE2K, SLC4A7 | CC | 0.0509 | 25 | 0.025974026 |

| | | | | | | | |
|---|---|---|---|---|---|---|---|
| GO:0044429 | mitochondrial part | 4 | SLC25A47, ACAT1, NLN, SLC25A27 | CC | 0.1302 | 27 | 0.027972028 |
| GO:0005813 | centrosome | 3 | PIBF1, MIB1, TUBGCP6 | CC | 0.3321 | 31 | 0.031968032 |
| GO:0044444 | cytoplasmic part | 23 | PIBF1, CLCN3, PRSS37, ANKRD28, PDS5A, UBXN7, SLC25A47, UBE2K, ACAT1, TMEM8B, STX18, NLN, MIB1, WARS, CUL5, SELENOO, EIF2A, TUBGCP6, ARHGEF7, SLC25A27, SLC4A7, NCOR1, SEC16A | CC | 0.0164 | 32 | 0.032967033 |
| GO:0005743 | mitochondrial inner membrane | 2 | SLC25A47, SLC25A27 | CC | 0.3911 | 37 | 0.037962038 |
| GO:0015630 | microtubule cytoskeleton | 4 | PIBF1, MIB1, TUBGCP6, NCOR1 | CC | 0.0863 | 41 | 0.041958042 |
| GO:0005819 | spindle | 2 | TUBGCP6, NCOR1 | CC | 0.0643 | 46 | 0.046953047 |
